# Synergistic removal of emerging contaminants using bacterial augmented floating treatment bed system (FTBs) of *Typha latifolia* and *Canna indica* for rejuvenation of polluted river water

**DOI:** 10.3389/fmicb.2025.1512992

**Published:** 2025-03-04

**Authors:** Vandan Patel, Shruti Sharma, Chirayu Desai, Bhavtosh Kikani, Datta Madamwar

**Affiliations:** ^1^Department of Biological Sciences, P. D. Patel Institute of Applied Sciences, Charotar University of Science and Technology (CHARUSAT), Anand, Gujarat, India; ^2^Department of Environmental Biotechnology, Gujarat Biotechnology University (GBU), Gandhinagar, Gujarat, India

**Keywords:** antibiotic resistance genes, bacterial community, emerging contaminants, floating treatment bed systems, pollutant removal, river water treatment

## Abstract

**Introduction:**

Floating Treatment Bed systems (FTBs) provide an effective approach to remove pollutants from the rivers. These systems consist of aquatic plants anchored on mats, which support the growth of microbial communities. Such a synergy between plants and microbes in FTBs plays a pivotal role to improve efficacy of river restoration strategies.

**Methodology:**

The effectiveness of the FTBs was evaluated for the rejuvenation of polluted water from the Mini River in Gujarat, India. These systems consisted of wetland plants, either *Typha latifolia* or *Canna indica*, which were augmented with the bacterial consortium VP3. Furthermore, the 16S *rRNA* gene amplicon sequencing approach identified the dominant bacterial communities and relative microbial community shifts within the FTBs. The presence of emerging contaminants, antimicrobial resistance genes, and pathogenic bacterial species in the untreated river water was evaluated, along with their reduction following treatment through FTBs. This analysis yielded important insights into the microbial dynamics governing the reduction of these contaminants.

**Results and discussion:**

The bacterial augmented FTBs consisting wet plants achieved reduction of 57%, 70%, 74%, and 80% in biochemical oxygen demand (BOD), chemical oxygen demand (COD), total phosphate, and sulfate, respectively. Moreover, the 16S *rRNA* gene amplicon sequencing identified *Proteobacteria* as the dominant phylum, with *Pseudomonas* species and *Hydrogenophaga* species being the most abundant genera in FTBs containing *T. latifolia* and *C. indica*, respectively. The functional gene prediction indicated presence of various xenobiotic degrading genes too. Non-targeted LC-HRMS analysis of treated water demonstrated complete elimination of antibiotic derivatives and dye intermediates, along with the partial removal of pharmaceutical and personal care products (PPCPs) and chemical intermediates. Additionally, the abundance of probable pathogenic bacteria and dominant antibiotic resistance genes was significantly reduced upon treatment. The phytotoxicity analysis of the treated water supported the outcomes. The studies on removal of emerging contaminants in the polluted river ecosystem has been relatively less explored, highlighting novelty and future possible applications of the plant-microbial augmented FTBs in rejuvenation of polluted rivers.

## Graphical Abstract

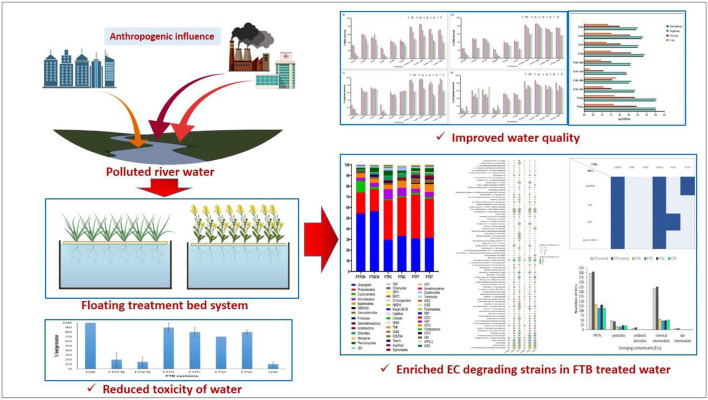

## 1 Introduction

The rivers are facing an unprecedented crisis as a result of rapid industrialization and anthropogenic activities globally (Gomes et al., 2023; Bandyopadhyay and De, 2017). Starting from the mighty Amazon to the Ganges, these crucial waterways that have nurtured life for centuries are now in the danger (Gomes et al., 2023; Su et al., 2023; Richards et al., 2023). This crisis has damaged the aquatic ecosystems and also poses health risks to billions of people depended on these waterways (Madhav et al., 2020).

These rivers are directly exposed to varieties of anthropogenic pollutants originating from industrial effluents, agricultural runoff, domestic sewage, and disposal of solid waste. Consequently, the number of severely polluted rivers in India is increasing at an alarming rate, posing substantial health risks to communities residing along their banks. The presence of highly toxic, carcinogenic, and mutagenic substances in these water bodies underscores the urgent need for eco-friendly, cost-effective, and sustainable mitigation strategies to address river pollution.

Biological treatment technologies have emerged as promising solutions to mitigate river pollution effectively (Singh et al., 2020). Amid, traditional phytoremediation techniques, such as constructed wetlands have shown superior pollutant removal efficiency and cost-effectiveness. However, their widespread adoption is hindered by significant land area they require. In contrast, floating treatment bed system have gained prominence as viable alternatives for restoring eutrophic rivers and lakes due to their demonstrated effectiveness and ease of management (Tao et al., 2020; Yadav and Goyal, 2022). Studies have highlighted its potential as an affordable technology for restoring polluted rivers, primarily due to its minimal land requirement and lower operational costs compared to other treatment methods (Yadav and Goyal, 2022; Asghar et al., 2022; Fahid et al., 2020; Tao et al., 2020).

The Floating Treatment Bed system (FTBs) represent a practical approach to enhance pollutant removal in rivers (Nandy et al., 2022; Liu et al., 2016; Samal and Trivedi, 2020; Huang et al., 2020; Yao et al., 2021). FTBs typically consist of aquatic plants and a specialized mat structure that supports an enhanced microbial population. This synergistic combination of plants and microbes plays a crucial role in improving the efficacy of river restoration strategies (McCorquodale-Bauer et al., 2023). However, despite their advantages, the application of FTBs for removing emerging contaminants from polluted river water remains underexplored. Rivers contaminated by diverse waste sources often carry emerging contaminants, posing challenges for effective river restoration (Srivastava et al., 2017).

Antibiotic resistance has emerged as a critical environmental and public health concern, affecting ecosystems, human health, as well as animal husbandry (Koch et al., 2021; Hernando-Amado et al., 2019). The origins of antibiotic resistance are multifaceted and complex, influenced by factors such as overuse and improper disposal of antibiotics in both human medicine and agriculture (Kinney and Heuvel, 2020; Bengtsson-Palme et al., 2018). Ávila et al. (2021) demonstrated the effective removal of antibiotics and antibiotic resistance genes (ARGs) from urban wastewater using vertical subsurface flow constructed wetlands, highlighting the potential of phytoremediation techniques in mitigating antibiotic resistance in the aquatic environments. Similarly, Bano et al. (2023) successfully treated amoxicillin-contaminated water using an improved FTB augmented with antibiotic-degrading bacteria, showcasing the capability of microbial technologies to address antibiotic pollution in water bodies.

The current study employed two native wetland plants, *Typha latifolia* (*T. latifolia*) and *Canna indica* (*C. indica*), cultivated on floating beds to investigate their efficacy in improving river water quality. The study monitored changes in physico-chemical parameters, such as biological oxygen demand (BOD), chemical oxygen demand (COD), total phosphate and sulfate along with pathogenic bacterial counts as indicators of water quality improvement. Additionally, 16S *rRNA* gene amplicon sequencing approach was used to identify the predominant bacterial communities within the FTBs, while the Shannon-Wiener diversity index characterized microbial community structures. The occurrence of antibiotics and corresponding ARGs was assessed in all FTBs at the end of the operational period, providing insights into the dynamics of microbial populations involved in antibiotic removal. This comprehensive approach lays a foundation for enhancing the overall quality of polluted urban rivers, offering valuable insights into the potential of FTBs as sustainable solutions for mitigating anthropogenic pollution and improving overall health of the invaluable riverine ecosystems.

## 2 Materials and methods

### 2.1 Collection and characterization of water from polluted river

The water was collected from the Mini river, Vadodara, Gujarat, India (22°23′35” N, 73°05′49” E). The Mini river receives pollutants from various sources, i.e., industrial, household as well as agricultural runoff. Samples were filtered using 0.45 μm nylon filter paper (PALL Corporation, USA) prior to analysis, and all the mentioned parameters were evaluated in triplicates. The parameters such as pH, EC, TDS, total nitrogen and dissolved oxygen (DO) were analyzed using HQ440D multi-meter (HACH, USA). The samples were analyzed for chemical oxygen demand (COD), biochemical oxygen demand (BOD_5_), total phosphate, and sulfate were estimated using closed reflux spectrophotometric method, titrimetric method, stannous chloride method, and turbidimetric method respectively as per American Public Health Association (APHA, 2017) for the characterization of the river water quality based on the Indian Standard Water Quality Guidelines (BIS, 2012).

### 2.2 Enrichment of bacterial consortium from soil sediment of industrially polluted sites

The bacterial consortia for bioremediation of the polluted Mini river water were developed following enrichment culture technique using polluted soil and sediments collected from the Mini river as shown in Table 1. A total of five bacterial consortia were developed and enriched in the polluted water of the Mini river supplemented with Bushnell-Hass medium (BHM) along with co-substrates (0.01% w/v glucose and 0.01% w/v yeast extract) at 37°C. The active cultures were sub-cultured on every 5^th^ day. After 50 subcultures the enriched bacterial consortia were evaluated for treatment of the polluted water of the Mini river by analyzing their treatment efficiencies for COD and BOD removal as described in the standard methods for water and wastewater analysis (APHA, 2017).

**Table 1:** Source of enriched bacterial consortia; where, GIDC stands for Gujarat Industrial Development Corporation.

**Consortia**	**Source**	**Location**
VP1	Wastewater polluted site	GIDC, Vatva, Gujarat
VP2	Waste disposal site	Sankarda, Gujarat
VP3	Wastewater polluted site	GIDC, Nandesari, Gujarat
VP4	Mini river	Anagadh, Gujarat
VP5	Mahi river	Sindhrot, Gujarat

### 2.3 Development of small-scale floating treatment bed systems

Specifically, six parallel laboratory scale FTBs were developed using plastic tanks (60 cm × 40 cm × 22.5 cm). The effective depth of the device was about 20 cm, and the total volume was 10 L. The FTBs were consisted of either of the two floating frameworks; (1) Styrofoam sheet or (2) Polyvinyl chloride pipe-frames (Supplementary Figure S1a). Each of the FTB developed with Styrofoam sheet has equally spaced eight PVC holders with the diameter of 6 cm. All the holders contain coconut coir as a floating bed material. Whereas, FTB developed with PVC pipe-frames have fiber mat with coconut coir as support (Supplementary Figure S1b). Two potential macrophytes, *T. latifolia* and *C. indica* originated from polluted zone of the Mini river, Gujarat, India were collected for the study. Both the plants were acclimatized in polluted river water for 21 days before actual experiments in FTBs. Each FTB contains total of 24 plant saplings. Detailed description of each FTB is enlisted in Table 2. The bacterial consortium, VP3 was enriched using the samples collected from the polluted source to capture the indigenous bacteria capable of removal of emerging contaminants. VP3 augmentation was performed for VP3-augmented FTBs taking 1L active culture of VP3 consortium. The consortium VP3 for augmentation was prepared using Bushnell Haas Medium (BHM) without the addition of external BOD. The inoculum was applied at a concentration of 10^7^ cells/mL, ensuring an effective microbial load for bioaugmentation. The developed FTBs were operated at different HRTs, mainly 1 day, 2 day, 3 day, 5 day, and 10 day.

**Table 2:** Floating treatment bed (FTB) systems used for the study; where, first two letters FT, Floating bed treatment; P, Polystyrene frame; S, styrofoam frame; CN, control without plant; CI, *C. indica*; TL, *T. latifolia*.

**Name of FTB**	**Floating mat**	**Floating matrix**	**Plant used**
FT-P-CN	Polystyrene pipes and nylon mesh	Coconut coir	No plant (control)
FT-S-CN	Styrofoam sheet and plastic pots	Coconut coir	No plant (control)
FT-P-CI	Polystyrene pipes and nylon mesh	Coconut coir	*C. indica*
FT-P-TL	Polystyrene pipes and nylon mesh	Coconut coir	*T. latifolia*
FT-S-CI	Styrofoam sheet and plastic pots	Coconut coir	*C. indica*
FT-S-TL	Styrofoam sheet and plastic pots	Coconut coir	*T. latifolia*

### 2.4 Performance evaluation of FTB

The quality of FTB treated polluted water was determined using various physico-chemical parameters, according to the standard methods for water and wastewater analysis (APHA, 2017) as described in the Section 2.1.

Pathogenic indicator bacterial populations of each FTB were determined using membrane filtration techniques (MFT) using different selective media such as Hi-Chrome M-TEC for *Escherichia coli*, deoxycholate citrate agar for *Salmonella* species and *Shigella* species, and TCBS media for *Vibrio cholerae*. All media for the study were purchased from Hi-Media, India. All samples were serially diluted for enumeration of bacterial pathogens using 0.9% (w/v) sterile normal saline. The samples were filtered using S-Pak white gridded filter membranes (Merck Millipore, Germany). The filter membrane was aseptically removed and placed over the agar surface of the respective agar plate. All the plates were incubated at 37°C for 24 h. Standard viable counting was performed to evaluate the colony-forming units (CFU/mL) representing the pathogens.

### 2.5 Determination of emerging contaminants removal by FTB from polluted river water

Removal of emerging contaminants from polluted water of the Mini river by FTB were analyzed based on the previously reported methods with few modifications (Liu et al., 2021; Zhou et al., 2012). The untreated and treated river water samples were subjected to solid-phase extraction (SPE) using Oasis HLB cartridges with specifications of 60 μm particle size, 80 Å pore size, 500 mg sorbent weight and 6 cc barrel size (Waters, Milford, MA, USA). Cartridges were pre-conditioned with 10 mL methanol, followed by 15 mL ultrapure water (3 times × 5 mL). Later, the untreated and treated river water samples were passed through the pre-conditioned SPE cartridges and elution was carried out using 10 mL methanol in the Waters Extraction Manifold (Waters, Milford, MA, USA). The extracts were concentrated and re-constituted in 10 mL ACN: H_2_O (9:1) and filtered through 0.2 μm filter before subjecting to LC-HRMS analysis. The extracted sample (10 μL) was injected using the in-built auto-sampler into a Thermo Scientific™ Hypersil GOLD™ C18 column [100 mm (l) × 2.1 mm (i.d.), 1.9 μm particle size] maintained at 30 °C at a flow rate of 0.3 mL/min. The mobile phase comprised of eluent A (0.1% formic acid in ultrapure water) and eluent B (acetonitrile with 0.1% formic acid). Subsequently, elution and detection of ECs were performed using Thermo Scientific™ Vanquish™ (liquid chromatography) coupled with Orbitrap Exploris™ 240 mass spectrometer (Thermo Fisher Scientific, USA). The mass spectrometry was conducted with a heated electrospray ionization (HESI) source operating in positive and negative ionization modes and data analysis was conducted using Thermo Scientific™ Xcalibur™ 4.4 software.

### 2.6 Microbial community analysis

All the VP3-augmented FTBs and control FTBs (FT-P-CN, FT-S-CN) were dismantled and the plant roots and matrix materials were vigorously mixed with water samples of respective FTBs to acquire complete microbial community of the system. The metagenomic DNA was extracted by filtering 1,500 mL sample through using 0.22 μm DURAPORE PVDF membrane filter (Merck Millipore, Germany). The filter pads were transferred aseptically to the extraction of the metagenomic DNA using XpressDNA Soil Kit (MagGenome, India) according to the manufacturer's protocol. The experiments were conducted in triplicates. The extracted metagenomic DNA quantity and quality was checked using Nanodrop 2000 (Thermo Fisher Scientific, USA) and 0.8% w/v agarose gel electrophoresis, respectively. Pooled metagenomic DNA of each VP3-augmented FTBs were outsourced for high-throughput amplicon sequencing of the V3–V4 regions of the 16S *rRNA* gene using Illumina NextSeq 2000 platform (MedGenome, India). All of the 16S *rRNA* gene sequence data obtained for the study was submitted to NCBI under Project ID: 1170026 Biosample: All of the 16S rRNA gene sequence data obtained for the study was submitted to NCBI under Project ID: 1170026 Biosample: SAMN44091849 (Sample VP3), SAMN44089104 (Sample FT-S-TL+VP3), SAMN44089103 (Sample FT-S-CI+VP3), SAMN44089102 (Sample FT-P-TL+VP3), SAMN44089101 (Sample FT-P-CI+VP3), SAMN44089100 (Sample FT-S-CN), and SAMN44089099 (Sample FT-P-CN).

Multiplexed paired-end reads (2 × 300 bp) generated on the Illumina platform were analyzed using The Quantitative Insights into Microbial Ecology 2 (QIIME 2) program version 2022.8 (Bolyen et al., 2019). The “demux summarize” plugin was employed to assess the read quality, followed by quality filtering, denoising to amplicon sequence variants (ASVs), and chimeric sequence removal via “dada2 denoise-paired” plugin, where reads with Q < 30 were removed (Callahan et al., 2016). Feature-IDs were mapped to sequences from the representative sequence file and feature-table obtained after “q2-dada2.” Taxonomy classification was performed with the “feature classifier” (classify-sklearn) plugin leveraging a naive Bayesian classifier trained on the latest version (2022.10) of the Greengenes database (McDonald et al., 2012). The classified taxonomies were visualized using the “taxa barplot” plugin in QIIME 2-view.

The functional metabolic capability of the VP3-augmented FTB was predicted using PICRUSt2 software (Douglas et al., 2020) according to the denoizing algorithms that enables phenotypic predictions via functional Enzyme Commission (EC) number (Kanehisa and Goto, 2000).

### 2.7 Effect of FTBs on antibiotic resistance profiles of Mini river water

Each metagenome was evaluated for the presence of four antibiotic resistance genes (ARGs): *sul1* and *sul2* (resistant to sulphonamides), *blaTEM* (resistant to beta-lactams), and *aac(6*′*)-Ib-Cr* (resistant to aminoglycosides). These ARGs were previously identified as abundant in the Mini river (Patel et al., 2024), and thus were selected for evaluating the antibiotic resistance in Mini river water treated by VP3-augmented FTBs along with control FTBs. The Supplementary Table S1 contains a list of the ARG-specific primers used during the investigation. The primer sequences were obtained from the previous studies (Park et al., 2006; Pei et al., 2006; Adegoke et al., 2020). The polymerase chain reaction (PCR) was conducted in triplicates along with no template control using SureCycler 8800 Thermocycler (Agilent Technologies, USA). 1.2% w/v Agarose gel electrophoresis was performed to determine the presence of ARGs. The PCR program was carried out as follows: initial denaturation at 94°C for 4 min, followed by 30 cycles of denaturation at 94°C for 40 s, annealing at 55°C for 45 s and extension at 72°C for 60 s; and final extension at 72°C for 5 min.

The Invitrogen™ PureLink™ Quick Gel Extraction Kit (Thermo Fisher Scientific, USA) was used to extract the PCR products for cloning. Subsequently, the purified DNA was cloned using the Mighty TA-Cloning Kit (Takara, Japan) according to the manufacturer's instructions. Cloned plasmids were isolated using Invitrogen™ PureLink™ Quick Plasmid Miniprep Kit (Thermo Fisher Scientific, USA). Quantification of cloned plasmids was performed using NanoDrop 2000 (Thermo Fisher Scientific, USA). Log dilution of cloned plasmids was used in RT-qPCR to prepare standard curves for each primer pair. Prior to use for final RT-qPCR analysis, each pair of primers was validated against each standard for product size and annealing temperature confirmation using normal PCR amplification and 1.2% agarose gel electrophoresis. Quantification of targeted ARGs was performed using the Stratagene Mx3005P (Agilent Technologies, USA) in triplicate experiments as per the user instructions. The RT-PCR analysis was conducted using PowerUp™ SYBR^®^ Green MasterMix (Thermo Fisher Scientific, USA). The RT-PCR program was carried out as follows: initial denaturation at 95°C for 3 min; followed by 40 cycles of denaturation at 95°C for 30 s, annealing at 55°C for 30 s, and extension at 72°C for 60 s; and final extension at 72°C for 5 min.

### 2.8 Phytotoxicity analysis

Phytotoxicity of FTBs treated and untreated Mini river water was performed using *Vigna radiata* plant seeds following the procedure described by Rane et al. (2014). In brief, 10 healthy *V. radiata* seeds were kept in 20 mL FTBs treated and untreated samples at an ambient temperature, where 20 mL distilled water served as a control. The seed germination percentage was calculated after 3 days using following equation according to Ghosh et al. (2023):


$$\begin{array}{l} \text{Seed}\;\text{Germination}\;\text{Percentage}\;\left(\text{SG}\%\right)\;=\\ (Number\;of\;germinated\;seeds/\;Total\;number\;of\;seeds)\;\times \;100 \end{array}$$

### 2.9 Statistical analysis

Statistical analysis was performed for all the dataset using GraphPad Prism 8 (GraphPad Software, San Diego, CA, USA). One-way ANOVA was used for the data analysis. Post-hoc analysis was conducted using Tukey's multiple comparison test to determine significant differences between the groups. A value of *p* < 0.05 was considered statistically significant.

## 3 Results

### 3.1 River water characterization

The overall quality of the Mini river water is mentioned in Table 3. Designated best use water quality criteria suggested by the Central Pollution Control Board (CPCB) (2018) of India (https://cpcb.nic.in) reveals that the obtained values for BOD and DO were Below-E. It means that the water is not suitable for drinking, outdoor bathing, fisheries or irrigation purposes. Moreover, the values of COD, sulfate and phosphate are also above the acceptance limits of general standards set by CPCB of India for industrial discharge.

**Table 3:** Water quality assessment of the Mini river with reference to the standard Indian water quality criteria.

**Water quality parameters**	**Mini river water**	Water quality status as per IS:10500^*^	General industrial discharge limits (CPCB^**^)
pH	8.62 ± 0.04	C	5.5–9.0
Temperature (°C)	27.5 ± 0.00	-	Not ≤ 5°C from AT
DO (mg/L)	1.55 ± 0.78	Below-E	-
COD (mg/L)	898.4 ± 25.35	-	250
BOD (mg/L)	35.27 ± 0.42	Below-E	30
Sulfate (mg/L)	872 ± 0.06	-	10
Phosphate (mg/L)	119.38 ± 1.98	-	5
Total Nitrogen (mg/L)	0.29 ± 0.02	-	0.3

^*^Indian Standard for drinking water specification. ^**^Central Pollution Control Board of India. C represents drinking water source after conventional treatment and disinfection, whereas Below-E represents not suitable for drinking, bathing or irrigation.

### 3.2 Performance evaluation of the enriched bacterial consortia

The degradation potential of the enriched bacterial consortia was evaluated based on the removal efficiencies for COD, BOD, sulfate, and phosphate. In total, 5 consortia were established (Table 1), of which VP3 performed best and was therefore used for bio-augmentation. The bacterial consortium VP3 demonstrated the following removal efficiencies (as shown in Table 4): COD (60.4 ± 4.14%), BOD (72.45 ± 0.1%), sulfate (57 ± 4.22%), and phosphate (75.89 ± 0.59%).

**Table 4:** Analysis of % removal efficiencies (±SD) regarding the potential of enriched bacterial consortia after 5days; where, COD, Chemical Oxygen Demand; BOD, Biochemical Oxygen Demand.

	**VP1**	**VP2**	**VP3**	**VP4**	**VP5**
COD	31.82 ± 9.88	23.43 ± 4.21	60.40 ± 4.13	44.55 ± 9.16	19.01 ± 2.57
BOD	63 ± 4.17	61.5 ± 2.69	72.45 ± 0.1	47.29 ± 0.51	55.02 ± 3.43
Sulfate	45.93 ± 1.18	37.19 ± 2.06	57.9 ± 4.22	41.06 ± 1.79	21.97 ± 3.59
Phosphate	70.9 ± 1.25	57.82 ± 1.08	75.89 ± 0.59	71.98 ± 1.04	53.27 ± 2.94

**Figure 1:**
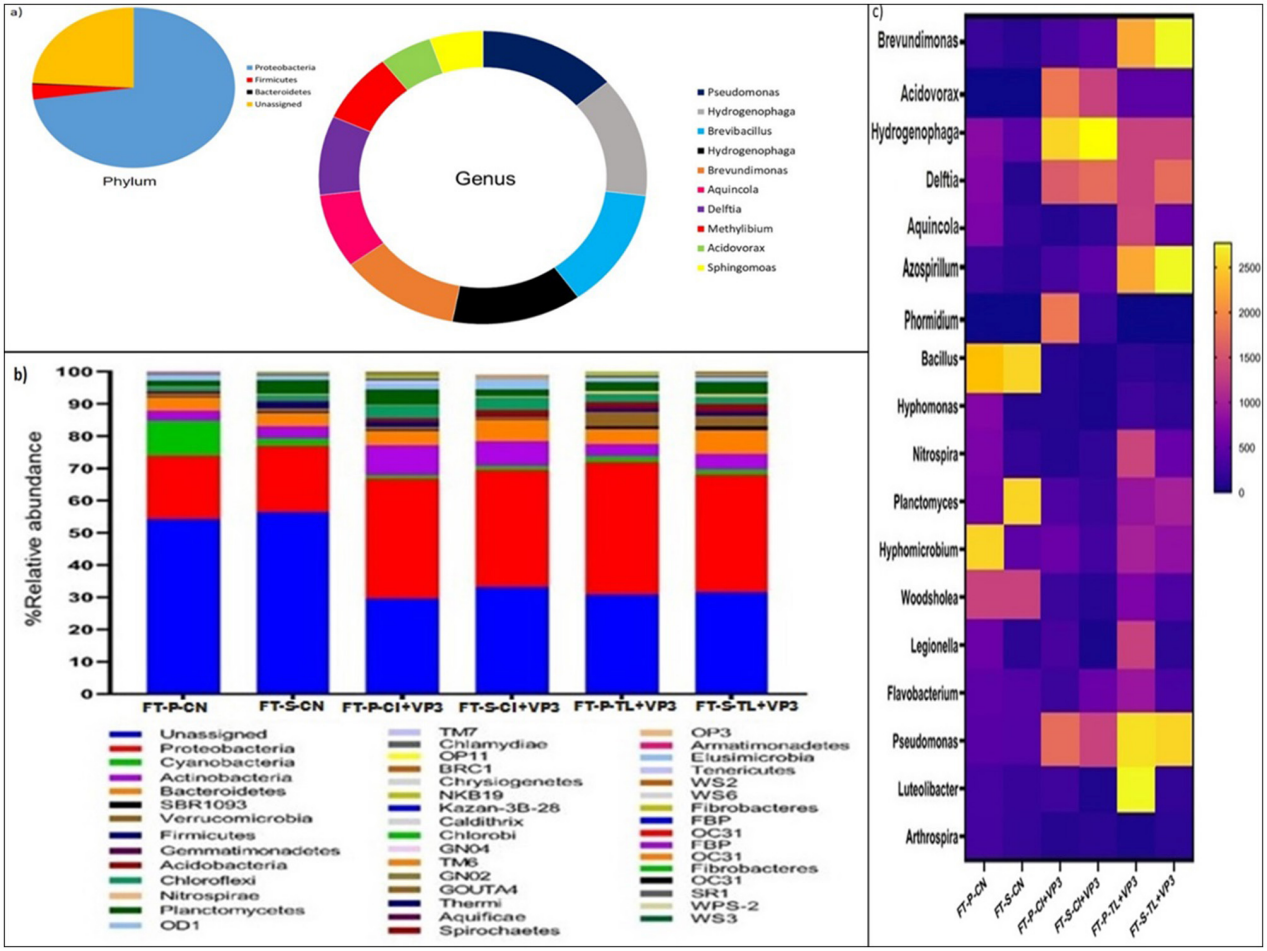
**(A)** Microbial community composition of the developed consortium VP3. Pie chart represents the phylum level microbial community and donut chart represents abundant genera of the consortium VP3. **(B)** Relative abundance of bacterial community at phylum level in VP3-augmented FTBs and FTB controls. FT, Floating bed treatment; P, Polystyrene frame; S, styrofoam frame; CN, control without plant; CI, *C. indica*; TL, *T. latifolia*. **(C)** Relative abundance of bacterial community at genus level in VP3-augmented FTBs and FTB controls. FT, Floating bed treatment; P, Polystyrene frame; S, styrofoam frame; CN, control without plant; CI, *C. indica*; TL, *T. latifolia*.

To characterize the microbial community of VP3, metagenomic DNA was extracted and sequenced. QIIME-2 analysis was performed to reveal the microbial community composition of VP3 (Figure 1A). The dominant phyla in VP3 were *Proteobacteria*, followed by *Firmicutes*, and *Bacteroidetes*. Where, the abundance of multiple genera namely *Pseudomonas, Hydrogenophaga, Brevibacillus, Brevundimonas, Aquincola, Delftia, Methylibium, Acidovorax*, and *Sphingomoas* belonging to different class of phylum *Proteobacteria* were obtained in consortium VP3.

### 3.3 Performance evaluation of the floating treatment bed systems

**Figure 2:**
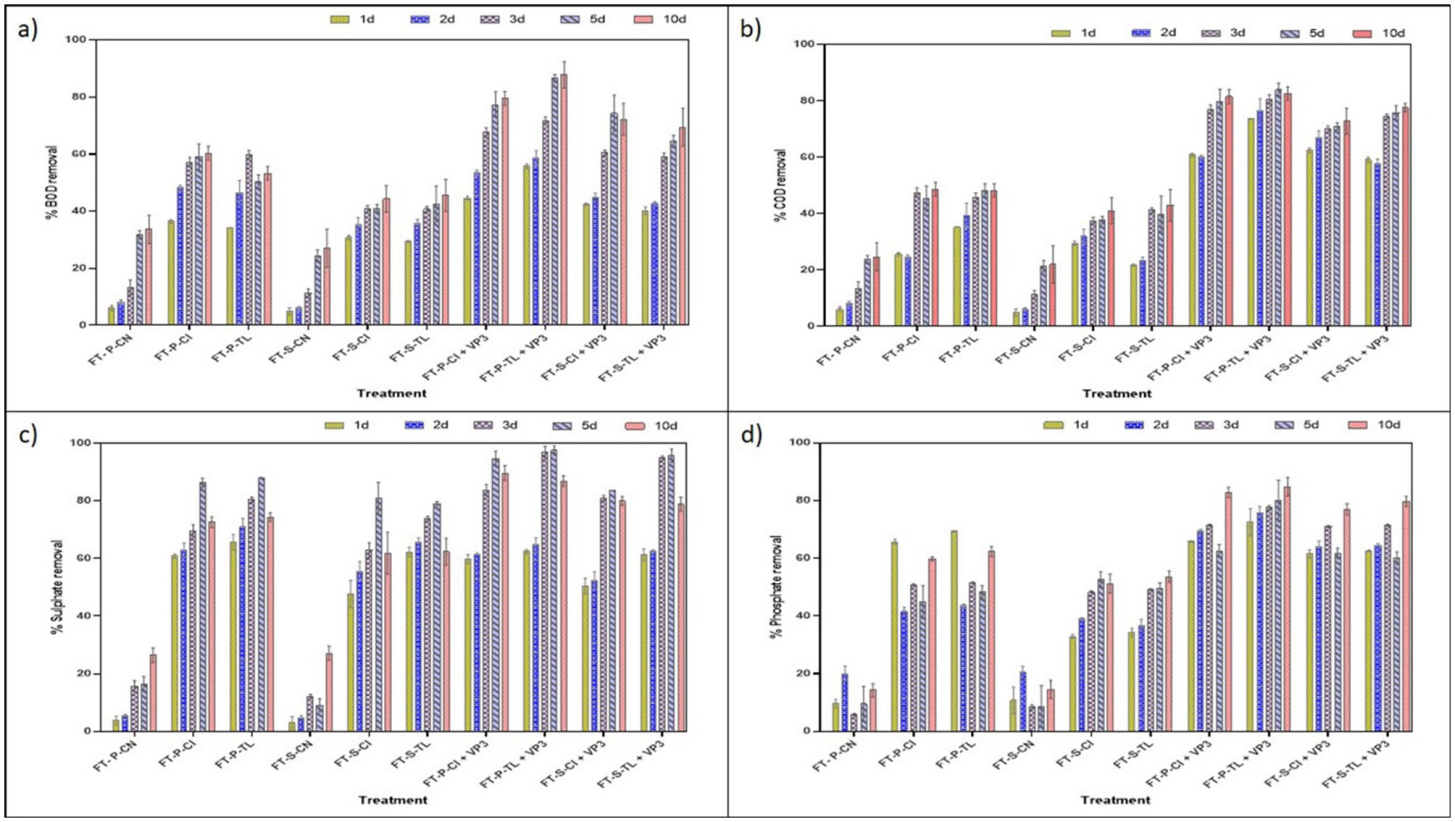
Performance evaluation of FTBs at different HRT **(A)** % BOD removal **(B)** % COD removal **(C)** % sulfate removal **(D)** % phosphate removal. FT, Floating bed treatment; P, Polystyrene frame; S, styrofoam frame; CN, control without plant; CI, *C. indica*; TL, *T. latifolia*.

The biological oxygen demand (BOD) and chemical oxygen demand (COD) are generally used to evaluate the organic and other pollutants in the waterbodies and their quality. In this study, VP3-augmented Floating Treatment Bed systems (FTBs) demonstrated significant performance, achieving over 57 ± 1.65% reduction in BOD (Figure 2A) and approximately 70 ± 1.34% reduction of COD (Figure 2B) in 3-day Hydraulic Retention Time (HRT). The performance of floating treatment beds (FTBs) was improved in presence of macrophytes, *T. latifolia* and *C. indica*. These plants achieved better remediation compared to the non-vegetated FTB controls (FT-P-CN and FT-S-CN), i.e., approximately 40 ± 1.65% BOD removal and 37 ± 1.67% COD removal at a 3-day hydraulic retention time (HRT). This highlights the role of macrophytes in improving the purification efficiency of FTBs. Moreover, sulfate and phosphate contributes significantly in the eutrophication process, generation of algal blooms, and oxygen depletion in the aquatic ecosystems. All vegetated floating treatment beds (FTBs), including FT-P-TL, FT-P-CI, FT-S-TL, and FT-S-CI, achieved over 60 ± 2.5% sulfate removal efficiency. Among these, VP3-augmented FTBs demonstrated the highest efficiency, with sulfate removal exceeding 80 ± 2.04% (Figure 2C). A similar pattern was observed for phosphate removal, with vegetated FTBs achieving an average efficiency of 51 ± 0.42% and the highest removal of 74 ± 0.6% recorded in VP3-augmented *T. latifolia*-planted FTBs (Figure 2D, Table S3).

### 3.4 Assessment of FTBs for the mitigation of the probable pathogens

**Figure 3:**
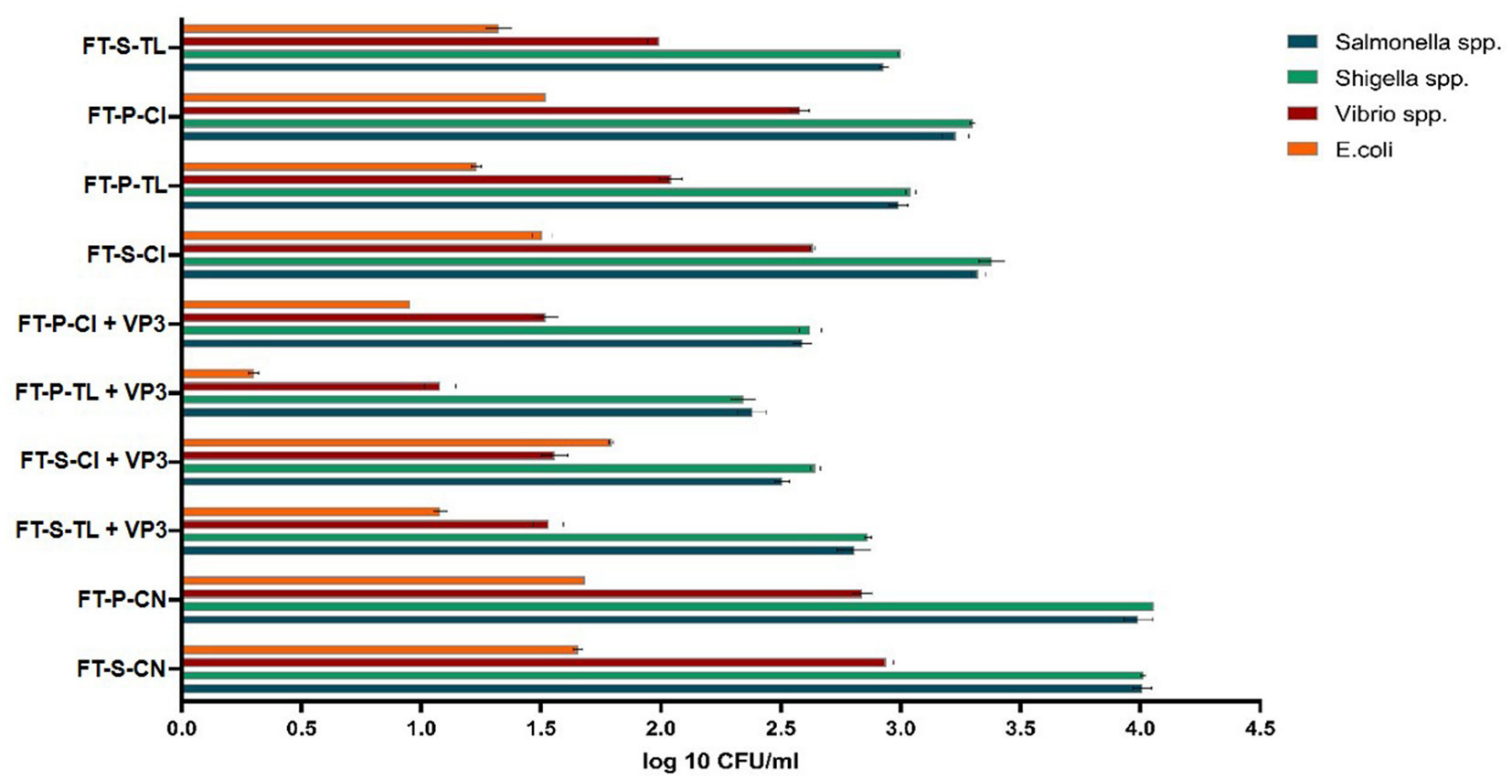
Probable pathogen abundance in Mini river water after FTB treatment. FT, Floating bed treatment; P, Polystyrene frame; S, styrofoam frame; CN, control without plant; CI, *C. indica*; TL, *T. latifolia*.

The effectiveness of VP3-augmented Floating Treatment Bed systems (FTBs) for the removal of microbial pathogenic strains was assessed using the selective plate count method. The control FTBs, which did not incorporate plants or the VP3 consortium, exhibited nearly twice as many pathogens compared to the experimental setups (Figure 3). Specifically, *Salmonella* and *Shigella* were found in the highest concentrations in the FT-P-CN (control FTB without plants and VP3) and were least prevalent in the FTBs containing VP3 and *T. latifolia* (FT-P-TL). Similarly, the counts for *Vibrio* species and *E. coli* were highest in FT-P-CN and FT-S-CN (control FTBs). The lowest pathogen counts were observed in the FTBs containing both VP3 and *T. latifolia*. This indicates that the combination of *T. latifolia* and VP3 is highly effective in reducing pathogen levels.

### 3.5 Evaluation of the microbial status of polluted Mini river water after FTB treatment

The microbial community structure of floating treatment beds (FTBs) was analyzed to assess the impact of the VP3 consortium on microbial composition. The predominant microbial phyla in VP3-augmented FTBs included *Proteobacteria, Actinobacteria, Bacteroidetes*, and *Planctomycetes*, demonstrating notable differences compared to control FTBs. The dominance of the *Proteobacteria* highlighted the significant influence of VP3 on the overall performance of the FTBs. In particular, the relative abundance of *Actinobacteria* increased to 7.2%, 6.4%, 3.1%, and 4.4% in FT-P-TL+VP3, FT-P-CI+VP3, FT-S-TL+VP3, and FT-S-CI+VP3, respectively, compared to 2.1% and 1.7% in the control FTBs FT-S-CN and FT-P-CN (Figure 1B). This phylum, previously associated with the bioremediation of heavy metals, antibiotics, and pesticides, exhibited enhanced abundance in VP3-augmented FTBs (Behera and Das, 2023; Antezana et al., 2023; Alvarez et al., 2017).

Additionally, increased relative abundances of *Acidobacteria, Chloroflexi*, and *Firmicutes* were observed in FT-P-CI+VP3 and FT-S-CI+VP3 compared to the controls. The phylum *Verrucomicrobia* was notably enriched in FT-P-TL (3.8%) and FT-S-TL (2.7%), suggesting plant-specific microbial community shifts. The *Proteobacteria* are known for their role in the bioremediation of xenobiotic pollutants, remained the most dominant phylum (Jokhakar et al., 2022; Srivastava and Verma, 2023; Zhang et al., 2020). *Firmicutes*, along with *Proteobacteria* and *Actinobacteria*, have been reported to establish compatibility with the rhizosphere of *T. latifolia* (Pietrangelo et al., 2018). Moreover, *Proteobacteria* were predominant in FTBs with *C. indica* for tetracycline antibiotic bioremediation (Xu et al., 2023). Notably, *Verrucomicrobia*, a key phylum in submerged membrane bioreactors for sulfonamide antibiotic removal, showed increased relative abundance in FTBs planted with *T. latifolia* (Yu et al., 2018).

At the genus level, microbial shifts were evident between control FTBs (FT-P-CN, FT-S-CN) and VP3-augmented FTBs (FT-P-CI+VP3, FT-S-CI+VP3, FT-P-TL+VP3, FT-S-TL+VP3), indicating the influence of VP3 and the planted macrophytes on microbial community composition. The control FTBs were dominated by the genera *Bacillus* species and *Woodsholea* species, whereas VP3-augmented FTBs with *T. latifolia* (FT-P-TL+VP3, FT-S-TL+VP3) exhibited a shift toward dominance of *Pseudomonas* species. In contrast, FTBs with *C. indica* (FT-P-CI+VP3, FT-S-CI+VP3) were predominantly enriched with *Hydrogenophaga* species (Figure 1C). The genus level microbial profiles in VP3-augmented FTBs closely mirrored those of the developed VP3 consortium. FTBs with *T. latifolia* were characterized by the dominance of *Pseudomonas* species, along with notable abundances of *Brevundimonas, Azospirillum, Hydrogenophaga, Delftia, Hyphomicrobium*, and *Planctomyces*. In contrast, FTBs with *C. indica* were primarily dominated by *Hydrogenophaga*, followed by *Delftia, Acidovorax, Pseudomonas*, and *Azospirillum*, showcasing plant-specific microbial community structures induced by the VP3 augmentation.

### 3.6 Functional gene annotation of the bacterial community involved in the FTB treatment

**Figure 4:**
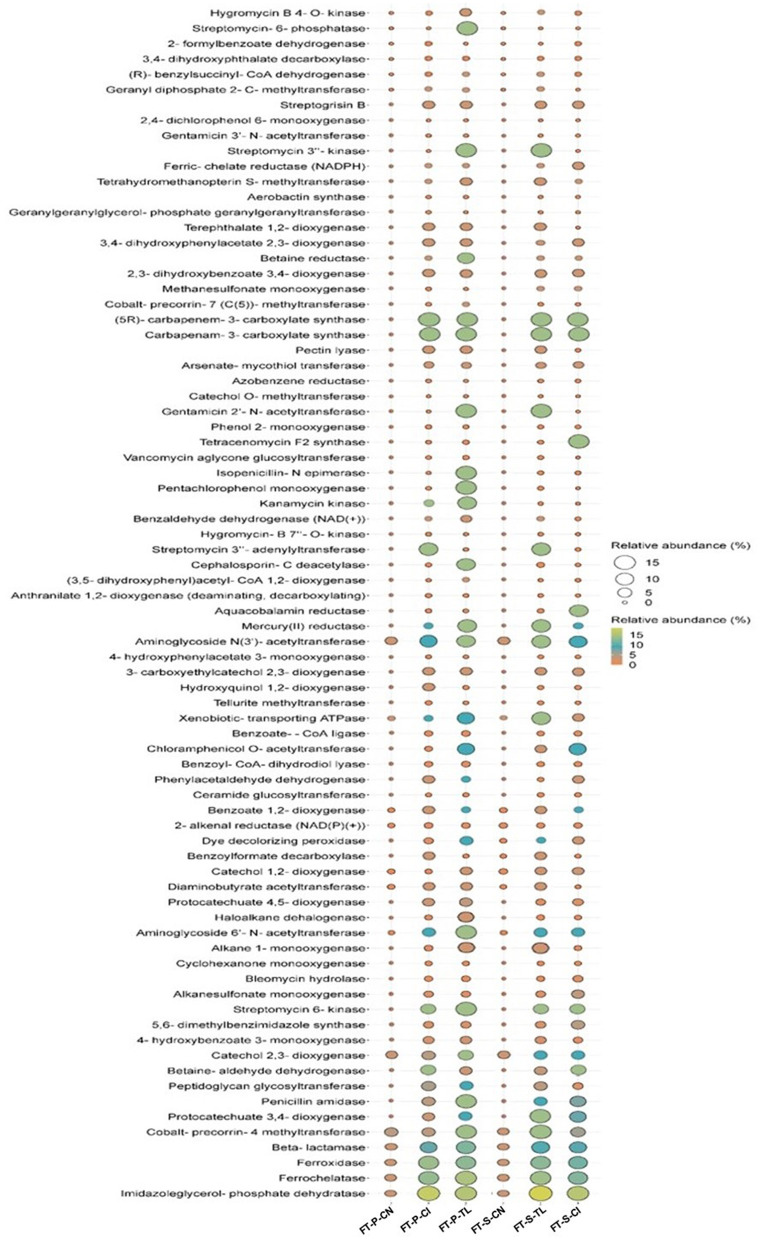
Predicted functional genes involved in biodegradation in VP3-augmented FTBs and FTB controls. FT, Floating bed treatment; P, Polystyrene frame; S, styrofoam frame; CN, control without plant; CI, *C. indica*; TL, *T. latifolia*.

The predicted metabolic capabilities acquired from PICRUSt based on 16S profiles of the FTB-treated samples identified 80 genes associated with the degradation of various xenobiotic compounds (Figure 4). Notably, the genes involved in antibiotic degradation were more prevalent in the test FTBs (FT-P-TL+VP3, FT-S-TL+VP3, FT-P-CI+VP3, FT-S-CI+VP3) compared to the control FTBs (FT-P-CN, FT-S-CN). Among these, approximately 40 genes were linked to the degradation of a range of pollutants, including antibiotics, pesticides, aromatic hydrocarbons, plasticizers, detergents, heavy metals, and azo dyes. The abundance of these genes was higher in the test FTBs (FT-P-TL+VP3, FT-S-TL+VP3, FT-P-CI+VP3, FT-S-CI+VP3) compared to the control FTBs (FT-P-CN, FT-S-CN). Conversely, 9 genes associated with antibiotic resistance were found in higher abundance in the control FTBs (FT-P-CN and FT-S-CN) and were present in lower quantities in the test FTBs (FT-P-TL+VP3, FT-S-TL+VP3, FT-P-CI+VP3, FT-S-CI+VP3). This suggests a reduced prevalence of antibiotic resistance mechanisms in the test FTBs compared to the controls. These findings indicate that VP3-augmented FTBs have a greater potential for the removal of various emerging contaminants.

### 3.7 Removal of emerging contaminants by FTB treatment

Non-targeted screening using LC-HRMS revealed the presence of various pollutants in the VP3-augmented FTBs after treatment. The analysis showed that FT-P-CN contained 304 pharmaceutical and personal care chemicals (PPCPs), which were reduced to 114 in FT-P-TL+VP3 and 132 in FT-P-CI+VP3. Similarly, FT-S-CN initially had 296 PPCPs, which were decreased to 112 in FT-S-TL+VP3 and 131 in FT-S-CI+VP3. Chemical intermediates, which are often associated with anthropogenic pollution and adverse effects on aquatic ecosystems were found in quantities of 226 and 217 in FT-P-CN and FT-S-CN, respectively. These numbers were significantly reduced in the VP3-augmented FTBs, with 49 intermediates in FT-P-TL+VP3, 47 in FT-S-TL+VP3, 51 in FT-S-CI+VP3, and 54 in FT-P-CI+VP3 (Supplementary Figure S2). Pesticides, known contributors to river pollution, were present in 44 compounds in FT-P-CN and 47 in FT-S-CN. The VP3-augmented FTBs reduced pesticide concentrations to 29 in FT-P-TL+VP3, 26 in FT-P-CI+VP3, 27 in FT-S-TL+VP3, and 24 in FT-S-CI+VP3. Notably, 7–12 antibiotic derivatives and 6 dye stuff intermediates were detected in FT-P-CN and FT-S-CN, but these compounds were completely eliminated in all VP3-augmented FTBs. The detailed information about various emerging contaminants have been provided in the Supplementary Table S2.

### 3.8 Impact of FTBs on antibiotic resistance profiles of the Mini river water

The results showed that the majority of the abundant ARGs of untreated Mini river water were absent in the FTBs treated samples (Supplementary Table S4). Specifically, the FT-P-CI+VP3 and FT-P-TL+VP3 completely eliminated all four ARGs. Whereas, FT-S-CI+VP3 and FT-S-TL+VP3 reduced the prevalence of these ARGs after treatment. However, the control FTBs; FT-P-CN and FT-S-CN revealed presence of all four tested ARGs. It indicates role of bacterial consortium and macrophytes in reduction of ARGs.

**Figure 5:**
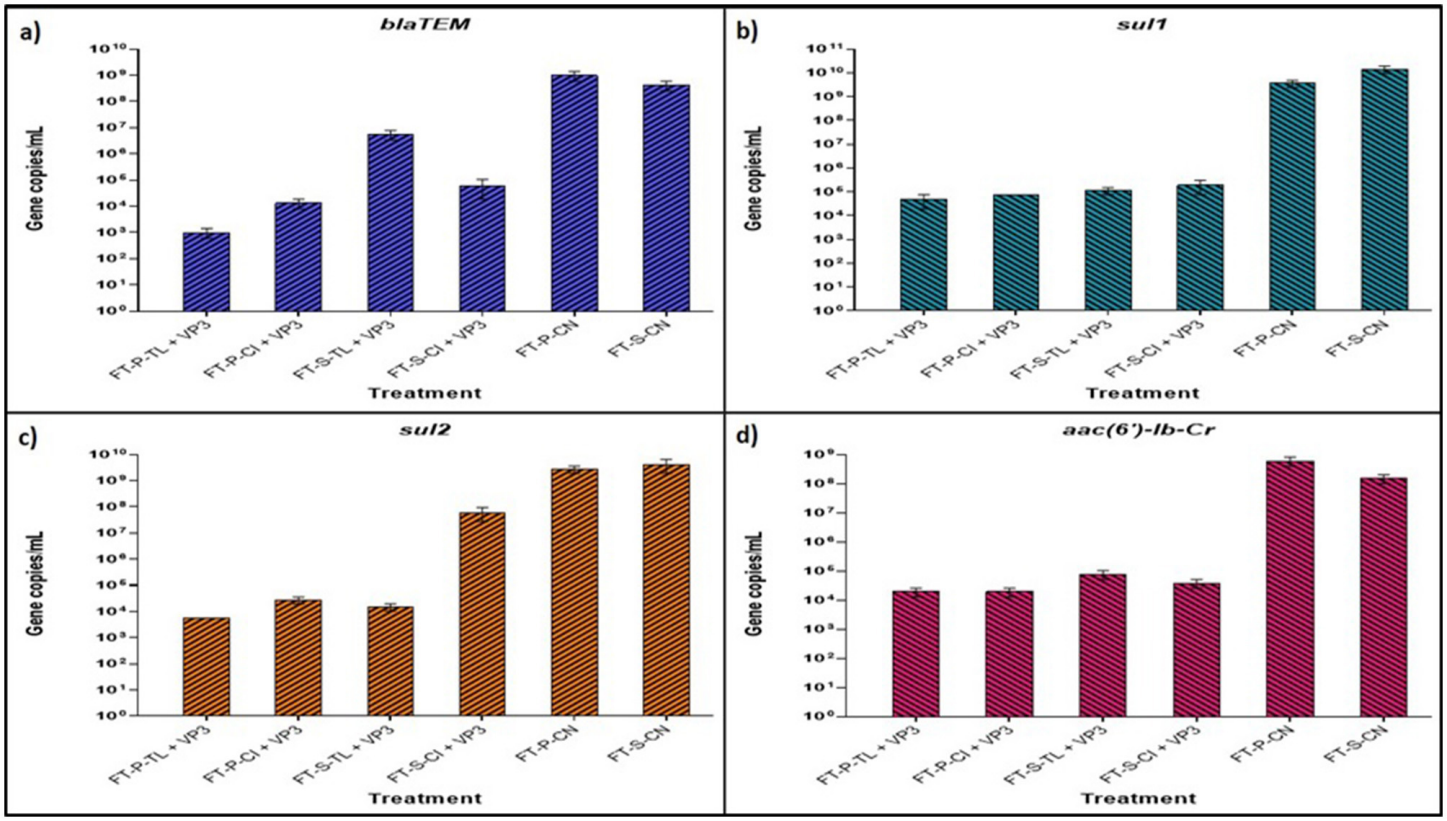
Absolute quantification of abundant ARGs using real-time qPCR of VP3-augmented FTB treated water samples **(A)** Absolute abundance of gene *blaTEM* **(B)** absolute abundance of gene *sul1* **(C)** absolute abundance of gene *aac (6*′*)-Ib-Cr* **(D)** absolute abundance of gene *sul2*. FT represents Floating bed treatment, P stands for Polystyrene frame, S stands for styrofoam frame, CN stands for control without plant, CI stands for *C. indica*, and TL stands for *T. latifolia*.

The Real-time PCR analysis of four selected antibiotic resistance genes (ARGs) confirmed a significant reduction in gene copy numbers following treatment with floating treatment beds (FTBs). The standard curves for the ARGs exhibited strong linear relationships (R^2^ > 0.925), and the melt curves validated the absence of non-specific amplification during RT-PCR (Supplementary Figure S3). Comparative analysis revealed a significant reduction in ARG abundance in FT-P-CI and FT-P-TL compared to control FTBs, FT-P-CN and FT-S-CN (Figure 5).

The *sul1* gene, conferring resistance to sulfonamide antibiotics, was the most prevalent ARG, with initial copy numbers of 3.57 × 10^9^ and 1.42 × 10^10^ copies/mL in FT-P-CN and FT-S-CN, respectively. These were substantially reduced to 4.72 × 10^4^, 7.55 × 10^4^, 1.13 × 10^5^, and 1.88 × 10^5^ copies/mL in FT-P-TL, FT-P-CI, FT-S-TL, and FT-S-CI, respectively. A similar trend was observed for *sul2*, which initially had 2.74 × 10^9^ and 4.22 × 10^9^ copies/mL in FT-P-CN and FT-S-CN, showing a reduction of 5.0 to 5.68 log folds in FT-P-TL, FT-P-CI, and FT-S-TL.

The aminoglycoside resistance gene *aac (6*′*)-Ib-Cr* showed a marked decrease from 6.04 × 10^8^ to 1.52 × 10^8^ copies/mL in the control FTBs to 1.91 × 10^4^ copies/mL in FT-P-CI and FT-P-TL. Similarly, the extended-spectrum beta-lactam resistance gene *blaTEM* exhibited significant reductions, with initial copy numbers of 9.99 × 10^8^ and 4.21 × 10^8^ in FT-P-CN and FT-S-CN dropping to 9.97 × 10^2^, 1.33 × 10^4^, 5.62 × 10^6^, and 6.2 × 10^4^ copies/mL in FT-P-TL, FT-P-CI, FT-S-TL, and FT-S-CI, respectively.

### 3.9 Phytotoxicity evaluation of the Mini river water treated using FTBs

The toxicity of Mini river water treated by VP3-augmented FTBs was evaluated using the *Vigna radiata* seed germination assay. The results indicated that the FTB planted in a PVC frame with *T. latifolia* was the most effective in reducing toxicity, followed by the FTBs with *C. indica* in PVC and Styrofoam frames. Water treated by the VP3-augmented FTBs demonstrated a higher percentage of seed germination compared to the controls FT-P-CN and FT-S-CN (Supplementary Figure S4). However, the percentage of seed germination in the treated water was slightly lower than that observed in the positive control (distilled water). Untreated Mini river water exhibited the lowest percentage of seed germination due to the presence of various pollutants.

## 4 Discussion

The Mini river water quality was not suitable for drinking as per the CPCB criteria. This adversely affects the riverine ecosystems and ultimately disturbs the food web. The FTBs vegetated with *T. latifolia* and *C. indica* in presence of VP3 efficiently reduced the COD and BOD of the polluted water of Mini river. This efficiency aligns with the known capabilities of floating treatments in handling organic pollutants. The VP3-augmented FTBs achieved approximately 70% COD removal within a hydraulic retention time (HRT) of 3 days. This aligns with previous studies demonstrating that microbial augmentation significantly enhances the biodegradation of organic and inorganic pollutants, achieving COD reductions of 65% to 85% depending on pollutant load and system design (Liu et al., 2021). Among the systems, FT-P-TL+VP3 achieved the highest phosphate removal of 100.1 ± 1.07 mg/L (~84.7 ± 0.91%) from an initial phosphate concentration of 118.1 ± 1.53 mg/L. The phosphate uptake by macrophytes such as *C. indica, T. latifolia, Phragmites australis*, and *Iris pseudacorus* has been previously reported to reduce nutrient levels in polluted wastewater due to their robust root systems, which support microbial growth and enhance phosphate uptake (Haritash et al., 2017; Han et al., 2022). Similarly, Chand et al. (2021) reported that constructed wetlands incorporating biochar and *Typha* species achieved 73.92% sulfate removal after a 6-day retention time. In this study, the VP3-augmented FTBs demonstrated a maximum sulfate removal of over 83.6% within a 5-day HRT, indicating the role of microbial mechanisms in bioremediation and facilitating nutrient uptake by macrophytes such as *T. latifolia* and *C. indica*. Kumar and Singh (2017) also reported that microbial consortium augmentation with *Eichhornia crassipes* enhanced sulfate removal efficiency in constructed wetlands. These findings confirm that VP3-augmented FTBs, including FT-P-TL+VP3, FT-P-CI+VP3, FT-S-TL+VP3, and FT-S-CI+VP3, exhibit superior pollutant removal potential compared to non-augmented FTBs (FT-P-CI, FT-P-TL, FT-S-CI, and FT-S-TL).

The effectiveness of VP3-augmented FTBs suggests a synergistic interaction between the bacterial consortium and the vegetated system. These findings are consistent with the earlier studies, such as Shahid et al. (2019); which reported improved water quality in FTBs vegetated with *Typha domingensis* and *Lepidagathis fusca*, augmented with a mixed bacterial culture for treatment of polluted water of Ravi river, Lahore. Where, they obtained the COD and BOD_5_ decreased to 47 and 21 mg/L from initial concentration of 405 and 190 mg/L, respectively in the system consisting bacteria augmented *T. domingensis*.

The probable pathogenic enumeration reveals that the inclusion of VP3 and *T. latifolia* in FTBs significantly reduces the abundance of microbial pathogens compared to control FTBs that lack these components. The significantly lower pathogen counts in the FTBs containing both VP3 and *T. latifolia* suggest a synergistic effect, where both components work together to improve the overall efficacy of the FTBs. The presence of *T. latifolia* likely contributes to enhanced pathogen removal through processes such as root filtration and microbial interactions, while the VP3 consortium may further assist in reducing pathogen counts through its bioremediation capabilities. This finding supports the use of VP3-augmented FTBs with macrophytes like *T. latifolia* in mitigating waterborne pathogenic outbreaks, especially in polluted waterways such as the Mini river. Earlier, Donde et al. (2020) emphasized the critical role of macrophytes in reducing fecal pathogenic bacteria and importance of combining local emergent and submerged macrophytes for efficient treatment. These finding uncovers the potential of VP3-augmented FTBs in managing microbial contamination in water bodies.

The microbial community of VP3 exhibits greater similarity with the microbial community at similarly polluted sites in Budha Nala and Tung Dhab drain at Punjab, India (Kumar and Saini, 2024). The phyla *Proteobacteria* and *Actinomycetota* consist of variety of genera efficient for bioremediation of polluted waterways (Joye et al., 2016) as well as potential plant growth promoting microbes involved in rhizo-remediation (Behera and Das, 2023; Singh et al., 2018). The phylum *Proteobacteria* comprises of genera such as *Brevundimonas* species, *Pseudomonas* species and *Desulfobacca* species reported for bioremediation of petroleum and oily sludge contaminated by total petroleum hydrocarbons, saturates, aromatics, and polar compounds (González-Toril et al., 2023). Chen et al. (2023) noted that a consortium dominated by *Pseudomonas* species, *Stenotrophomonas* species, and *Delftia* species exhibited high degradation potential for phenanthrene. This suggests that the microbial community within consortium VP3 has a robust capacity to metabolize a range of emerging pollutants.

To understand the potential of the FTBs, the composition of microbial communities developed during treatment is crucial. In this study, the dominant phyla *Proteobacteria, Actinobacteria, Bacteroidetes*, and *Planctomycetes* were consistent across all FTB configurations, indicating their fundamental role in the treatment processes. The microbial community of the VP3-augmented FTBs was dominated by phylum *Proteobacteria* representing the successful augmentation of the VP3 with the macrophytes used for the study. This consistent dominance of *Proteobacteria* in all the VP3-augmented FTBs reinforces potential of VP3 consortium for degradation of various pollutants (Jokhakar et al., 2022; Srivastava and Verma, 2023; Zhang et al., 2020). However, the control FTBs (FT-P-CN and FT-S-CN) where no plants were introduced has relatively less abundance of the phylum *Proteobacteria*. The increase in the relative abundance of *Acidobacteria, Chloroflexi*, and *Firmicutes* in FTBs with *T. latifolia* and *C. indica* suggests that these plant species may enhance the growth of these microbial groups, potentially improving bioremediation efficacy. Observed presence of *Actinobacteria* aligns with its ability to remediate heavy metals and organic pollutants (Antezana et al., 2023; Alvarez et al., 2017). Earlier, Pietrangelo et al. (2018) noted that the phyla of *Proteobacteria, Actinobacteria*, and *Firmicutes* involved in the foundation of the microbiota with some other phyla such as *Acidobacteria, Bacteroidetes, Chloroflexi*, and *Verrucomicrobia* which varies according to the plant species. The presence of *Firmicutes* in association with *T. latifolia* and its compatibility with rhizosphere environments supports its role in effective bioremediation within VP3-augmented FTBs with *T. latifolia*. Similarly, the prominence of *Proteobacteria* in FTBs with *C. indica* highlights its adaptability and efficacy in various bioremediation contexts. This combination of *Proteobacteria* with *C. indica* was earlier reported for degradation of tetracycline antibiotics from water using mycorrhizal fungi based vertical flow constructed wetlands with more than 90% removal efficiency (Xu et al., 2023). The increased abundance of *Verrucomicrobia* in FTBs planted with *T. latifolia* is compelling interest, as this phylum has been previously associated with the removal of sulfonamide antibiotics (Yu et al., 2018). This suggests that *T. latifolia* may foster conditions conducive to the growth of *Verrucomicrobia*, enhancing the overall pathogen removal efficiency of the FTBs.

These findings indicate that both plant species, *T. latifolia* and *C. indica*, play significant roles in shaping microbial community structure and enhancing the bioremediation capacity of FTBs. The synergy between plant and microbial components in FTBs discloses the potential for improved water treatment of polluted rivers. The prevalence of the genus *Pseudomonas* in FTBs with *T. latifolia* aligns with its well-documented role in biodegrading a wide range of pollutants, including antibiotics, personal care products (PPCPs), pesticides, heavy metals, and aromatic hydrocarbons (Tang et al., 2024; Pápai et al., 2023). The dominance of potential plant growth promoting bacterial genus *Azospirillum* in the VP3-augmented FTBs planted with *T. latifolia* enhances the stress tolerant abilities of the plant. Earlier genus *Azospirillium* was reported to have significantly reduced the damage to roots of *Polygonum hydropiperoides* planted constructed wetlands exposed to the heavy metal contamination (Barbosa et al., 2023). The presence of the Genus *Hydrogenophaga* species in FTBs with *C. indica* is also significant, as this genus is known for its ability to degrade aromatic compounds and certain chlorinated compounds used in pesticides and PPCPs (Borah et al., 2023; Yang et al., 2023). These findings unveil the impact of macrophytes on shaping microbial communities and highlight the role of specific bacterial genera in the bioremediation processes within FTB systems. The interplay between macrophytes and microbial communities are key contributors optimizing the effectiveness of pollutant removal in FTBs.

The effective degradation of xenobiotics is essential for mitigating their environmental impact and preventing the proliferation of antibiotic-resistant bacteria (ARB). The identification of approximately 40 genes related to the degradation of various pollutants including pesticides, aromatic hydrocarbons, plasticizers, detergents, heavy metals, and azo dyes, demonstrates the versatility of the test FTBs in addressing a wide spectrum of contaminants. These findings, supported by LC-HRMS analysis, confirm that VP3-augmented FTBs are highly effective in degrading critical pollutants such as antibiotics and dyes. Overall, the results underscore the strong potential of VP3-augmented FTBs to reduce diverse contaminants, making them valuable tools for improving water quality and addressing pollution challenges in river systems. The potential transfer of ARGs to pathogenic bacteria remains a significant global public health concern. However, the present study revealed a substantial reduction in ARG absolute abundances (1.84 to 6 log fold) within the VP3-augmented FTBs. The observed reduction in gene copies/mL highlights the progressive elimination of ARGs across these systems. For instance, Porras-Socias et al. (2024) reported a decrease in *sul1* abundance from 4.3 × 10^6^ to 6.9 × 10^4^ copies/mL in constructed wetlands planted with *Sparganium erectum*. Similarly, in this study, the VP3-augmented FTBs significantly reduced ARGs, including *sul1, sul2, blaTEM*, and *aac(6*′*)-Ib-Cr*, through mechanisms such as plant uptake, bacterial host die-off, or sorption to organic matter (Sabri et al., 2021). The robust root systems of wetland plants like *T. latifolia* and *C. indica* play a pivotal role in this process by supporting microbial communities involved in filtration, adsorption, absorption, and biotransformation of ARGs. Additionally, macrophytes provide oxygen to microbes and act as primary filters for solid particles, enhancing microbial activity, reducing ARG accumulation, and promoting overall ARG removal (Fang et al., 2017). These findings align with Donde et al. (2020), who demonstrated the effectiveness of *T. latifolia* in constructed wetlands for the removal of 12 antimicrobial agents and their associated resistance genes, achieving a purification efficiency of 75% in effluents from Lake Dianchi, China. Similarly, Ávila et al. (2021) reported that subsurface flow constructed wetlands vegetated with *Phragmites australis* effectively removed antibiotics, including ciprofloxacin, ofloxacin, pipemidic acid, and azithromycin, alongside ARGs such as *sul1* (46%−97%), *sul2* (33–97%), *ermB* (9%−99%), *qnrS* (18%−97%), and *blaTEM* (11%−98%). These studies support the current findings, emphasizing the significant role of the VP3 microbial consortium, combined with suitable macrophytes, in mitigating antibiotic resistance in river systems. This integrated bioremediation strategy highlights its potential to address complex pollution challenges in environmental remediation effectively.

The findings from the *Vigna radiata* seed germination assay suggests that the VP3-augmented FTBs effectively reduce the toxicity of Mini river water. Specifically, the FTBs planted with *T. latifolia* in PVC frames showed the highest efficiency in toxicity removal, making it the most promising option for further applications. The treated water from the VP3-augmented FTBs exhibited significantly higher seed germination rates compared to the untreated controls (FT-P-CN and FT-S-CN), although it did not fully match the germination rates seen with distilled water. These results are consistent with previous studies, such as those by Kumar et al. (2018), who reported that polluted Hindon river water adversely affected *Vigna radiata* seed germination, indicating poor water quality due to anthropogenic pollution. Their study concluded that diluted water was more suitable for irrigation practices. In contrast, the treated water from the VP3-augmented FTBs, particularly with *T. latifolia*, showed that it could be used directly without further dilution for agricultural purposes. Overall, the data highlighted the effectiveness of the VP3-augmented FTBs in removing toxicity from polluted water bodies, suggesting a viable approach for improving water quality and suitability for agricultural use.

## 5 Conclusion

The study demonstrates that the plant-microbial combination enhances the overall efficiency of floating treatment bed (FTB) systems in removing sulfate, phosphate, COD, and BOD from polluted river water. Further, it also exhibited significant potential for pathogen elimination from contaminated water. The metagenomic analysis revealed that the VP3-augmented FTBs host a microbial community predominantly composed of the genera *Pseudomonas* species and *Hydrogenophaga* species, both belonging to the phylum *Proteobacteria*. In addition, the VP3-augmented FTBs possess a range of functional genes critical for the degradation of antibiotics, pesticides, pharmaceutical and personal care products (PPCPs), polycyclic aromatic hydrocarbons (PAHs), and other emerging contaminants (ECs). ARG detection confirmed that VP3-augmented FTBs were more efficient in reduction of ARGs, with lower ARG distribution compared to control FTBs. It could remove 7–12 antibiotic residues, 6 dye intermediates, and over 50% of pesticides, which further establishes its effectiveness in reduction of ECs in the polluted rivers. Phytotoxicity testing using *Vigna radiata* revealed that water treated by FT-P-TL and FT-P-CI exhibited improved seed germination rates, indicating enhanced environmental safety. These findings highlight the potential of FTBs, especially those enriched with bacterial consortia, as effective and sustainable solutions for removing a broad range of emerging pollutants under environmental conditions. The study underscores the advantages of FTBs over intensive treatment approaches for improving water quality in polluted river systems.

## Data Availability

The datasets presented in this study can be found in online repositories. The names of the repository/repositories and accession number(s) can be found in the article/Supplementary material.
