# Supplementary material for: Synergistic removal of emerging contaminants using bacterial augmented floating treatment bed system (FTBs) of Typha latifolia and Canna indica for rejuvenation of polluted river water
Source: Front Microbiol. 2025 Mar 4;16:1512992. doi: 10.3389/fmicb.2025.1512992 (PMC11919279; doi:10.3389/fmicb.2025.1512992)
Supplement: Supplementary file 1 [file Data_Sheet_1.docx]

**Synergistic removal of emerging contaminants using bacterial augmented Floating Treatment Bed system (FTBs) of *Typha latifolia* and *Canna indica* for rejuvenation of polluted River water**

**Vandan Patel^a^, Shruti Sharma^a^, Chirayu Desai^b^, Bhavtosh Kikani^a^**^*^**, Datta Madamwar^a^**^*^

**SUPPLEMENTARY FILE**

**Figure S1** Designing details of Floating Treatment Bed systems (FTBs) and experimental setup. a) Details of floating frame used for FTBs. b) Designing descriptions of FTBs c) Experimental setup of designed FTBs.


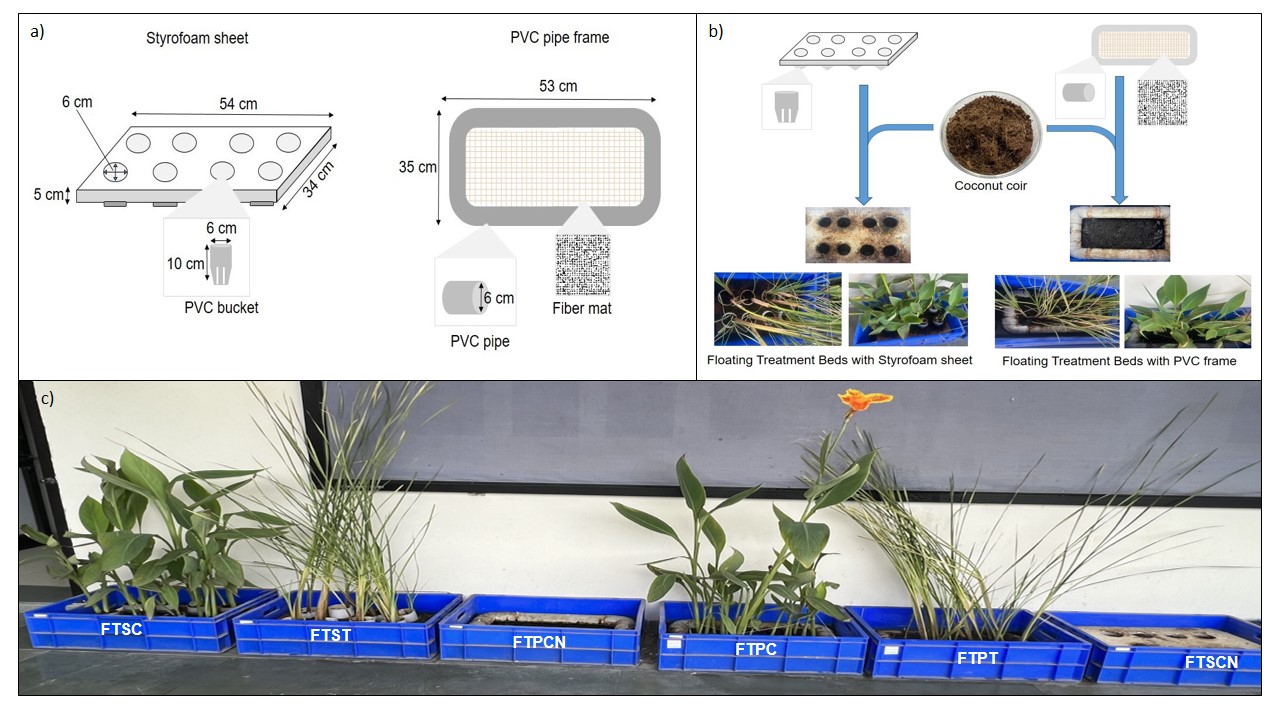


**Figure S2** Pollutant status of the Mini river water in VP3-augmented FTBs and FTB controls after treatment. FT represents Floating bed treatment, P stands for Polystyrene frame, S stands for styrofoam frame, CN stands for control, CI stands for *C. indica*, and TL stands for *T. latifolia*.

**
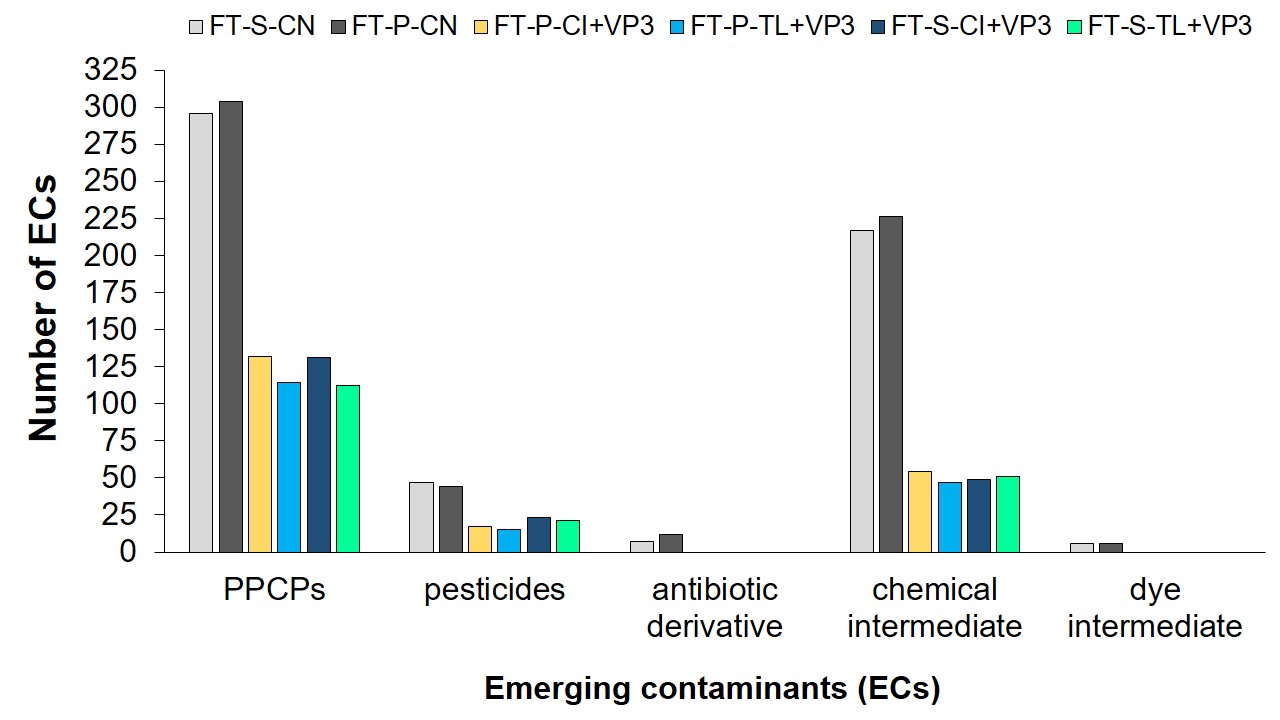
**

**Figure S3.** Standard cycle threshold values vs. copy number graph of the targeted ARGs and melt curve of the RT-PCR using respective ARG specific primers.

**
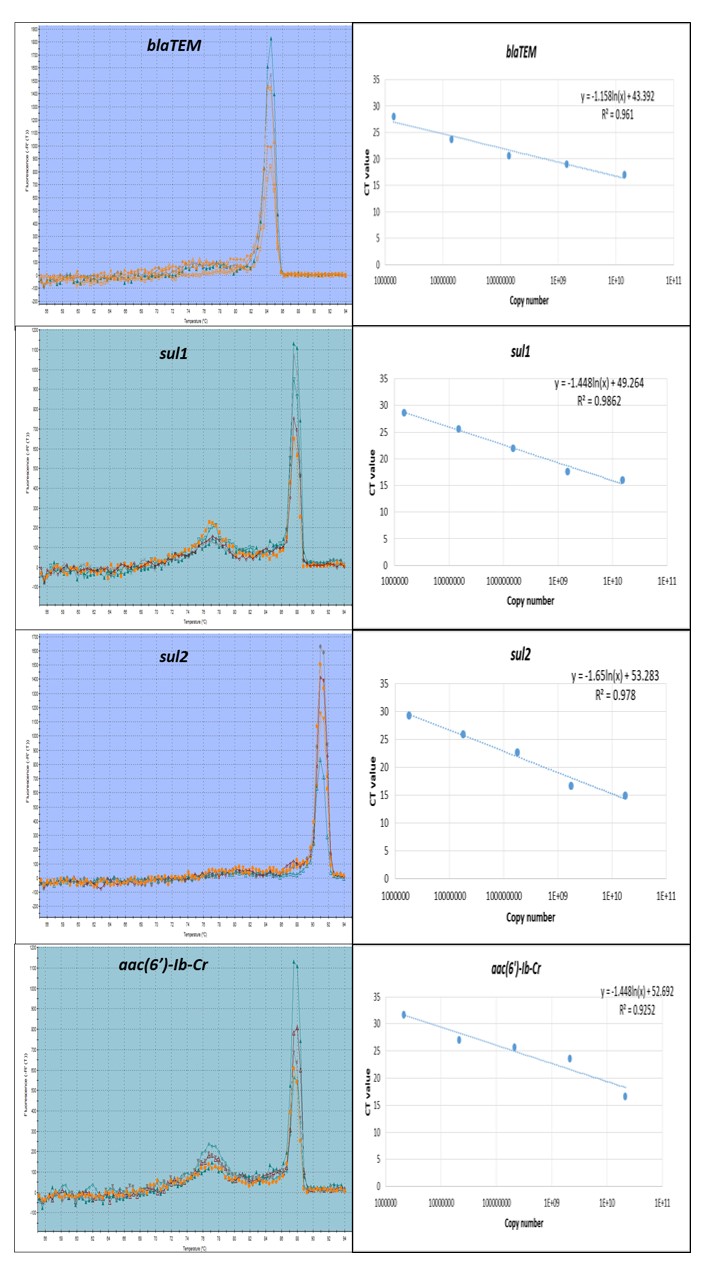
**

**Figure S4.** Toxicity analysis of VP3 augmented FTB treated water on *Vigna radiata.* Where, DW represents % seed germination in distilled water. UW represents % seed germination in untreated water of the Mini river. FT stands for Floating bed treatment, P stands for Polystyrene frame, S stands for styrofoam frame, CN stands for control without plant, CI stands for *C. indica*, and TL stands for *T. latifolia*. The significant p-value of <0.05 was considered for all the dataset (n=3).

**
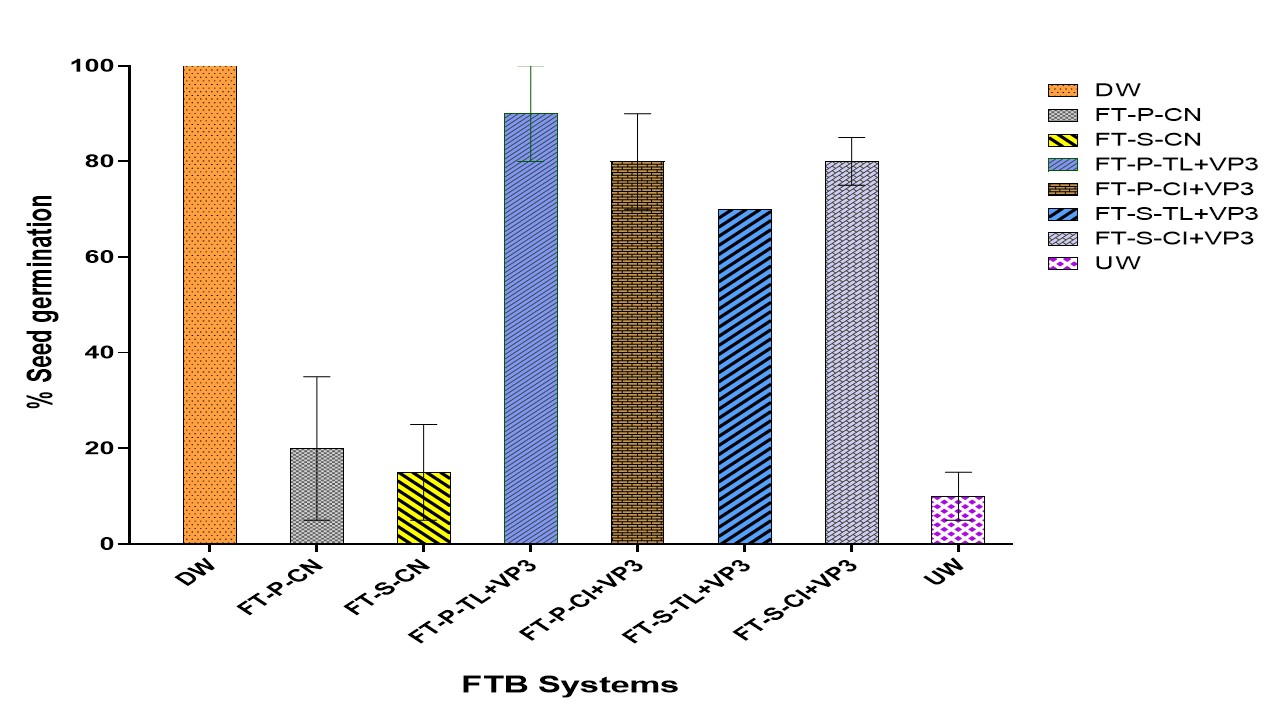
**

**Figure S5.** Comprehensive LC-HRMS analysis chromatogram for Non-targeted Pollutant Profiling of FTB treated Mini river water.


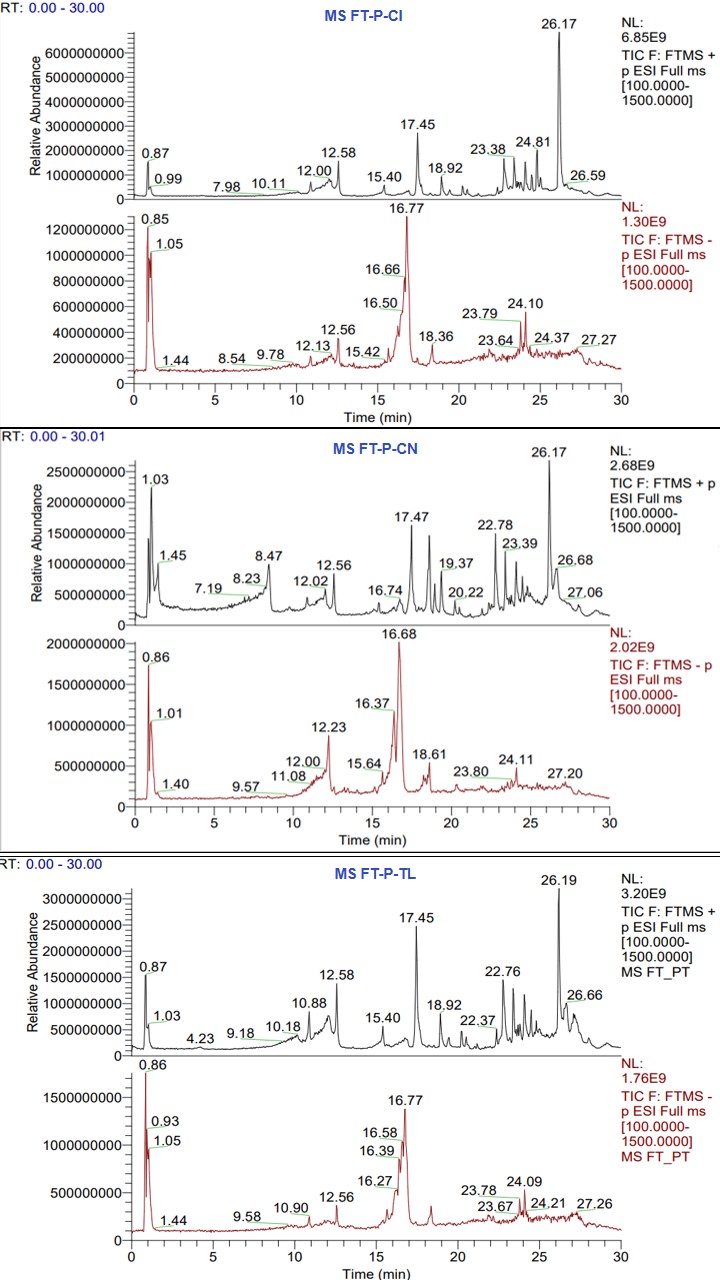


**
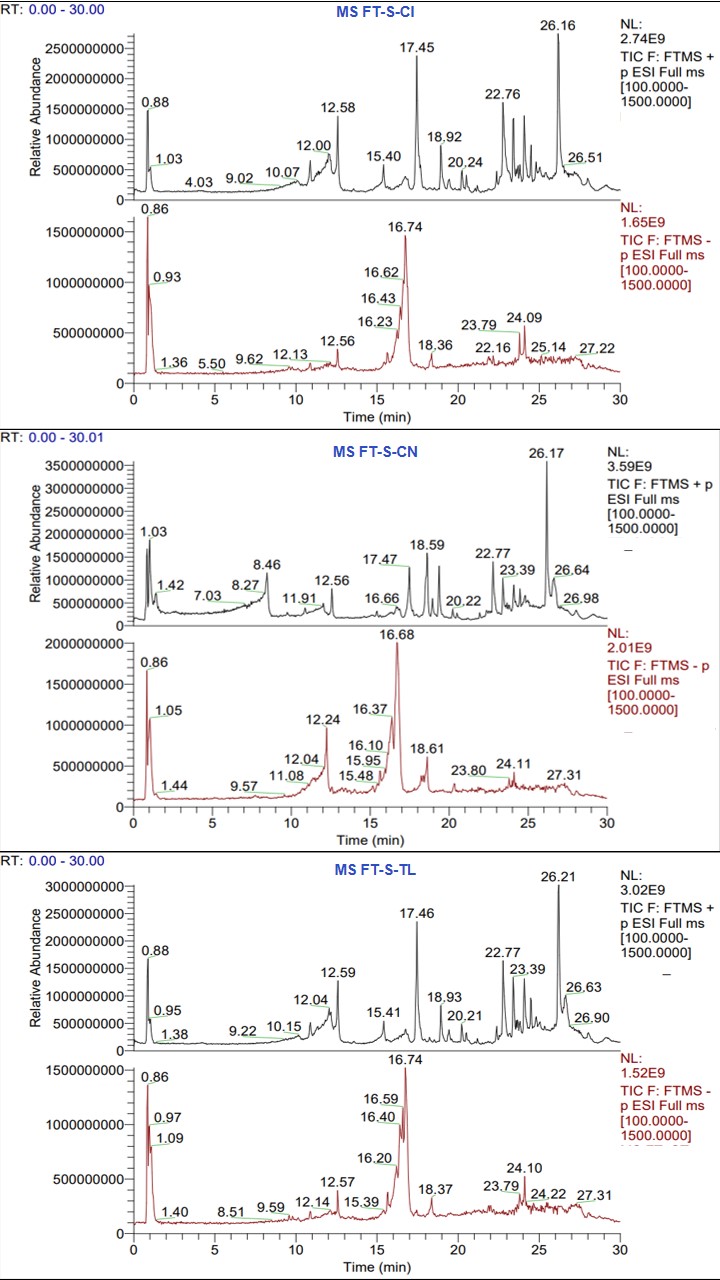
**

**Figure S6.** Comprehensive LC-HRMS analysis TIC for Non-targeted Pollutant Profiling of FTB treated Mini river water.

**
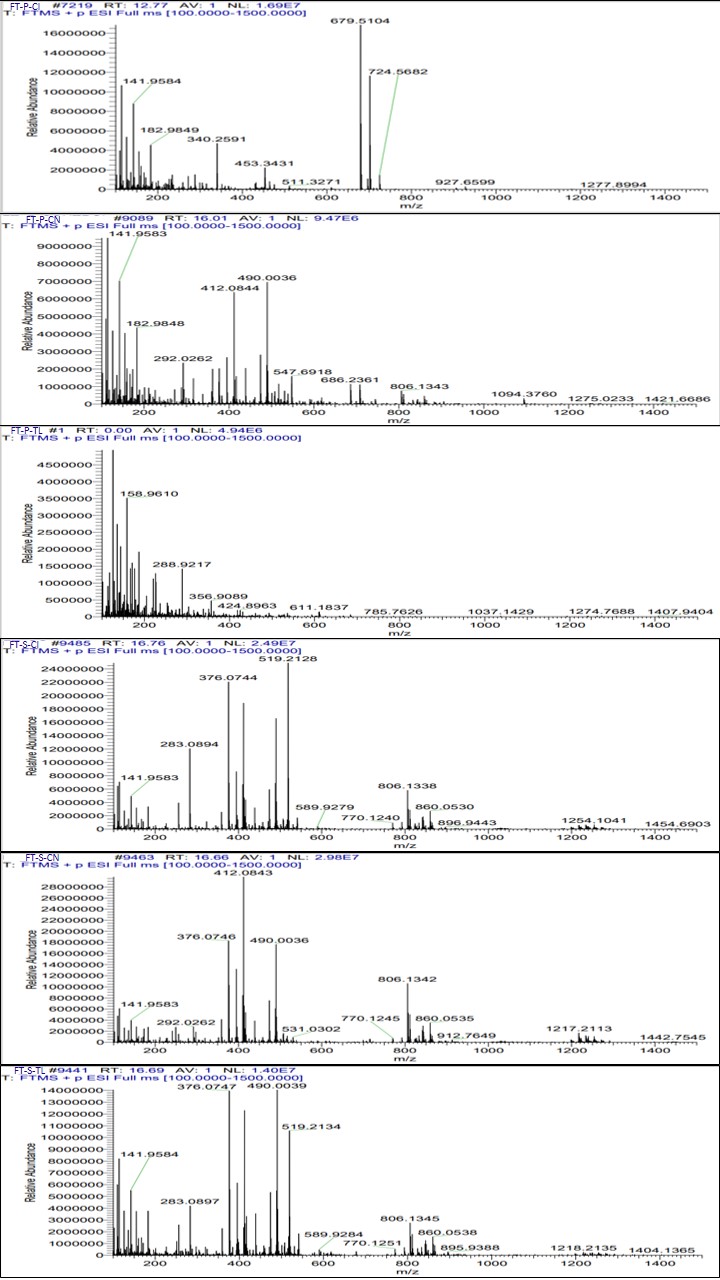
**

**Figure S7.** Growth curve of the bacterial consortium VP3 in the polluted water

**Table S1.** List of ARG specific primers. Where, FP stands for forward primer and RP stands for reverse primer.

| **Target gene** | **Amplicon size (bp)** |  | **Primer sequence (5’- 3’)** | **Antibiotic family** | **Reference** |
| --- | --- | --- | --- | --- | --- |
| *blaTEM* | 258 | FP | GTCGCCGCATACACTATTCTCA | β-lactam  ESBLS genes | Adegoke et al., 2020 |
|  |  | RP | CGCTCGTCGTTTGGTATGG |  |  |
| *sul1* | 163 | FP | CGCACCGGAAACATCGCTGCAC | Sulphonamides | Pei et al., 2006 |
|  |  | RP | TGAAGTTCCGCCGCAAGGCTCG |  |  |
| *sul2* | 190 | FP | TCCGATGGAGGCCGGTATCTGG | Sulphonamides | Pei et al., 2006 |
|  |  | RP | CGGGAATGCCATCTGCCTTGAG |  |  |
| *aac (6`)-Ib-cr* | 482 | FP | TTGCGATGCTCTATGAGTGGCTA | Aminoglycoside | Park et al., 2006 |
|  |  | RP | CTCGAATGCCTGGCGTGTTT |  |  |

The code FP was given to the forward primer, whereas the code RP was given to the reverse primers

**Table S2.** A comparative analysis about removal of various classes of ECs after the rejuvenation of water, as evaluated by LC-HR/MS

| **Category** | **Name of the compound** | **FT-P-CN** | **FT-S-CN** | **FT-P-CI** | **FT-P-TL** | **FT-S-CI** | **FT-S-TL** |
| --- | --- | --- | --- | --- | --- | --- | --- |
| **Antibiotic derivatives** | 1-methyl-4-ethyl formate-5-pyrazole sulfonamide | Present | Absent | Absent | Absent | Absent | Absent |
|  | 2-{2-[(Chloromethyl)sulfonyl]-2-(5-nitro-2-furyl)vinyl}-5-phenylfuran | Present | Present | Absent | Absent | Absent | Absent |
|  | 3,3-Dimethyl-7-oxo-4-thia-1-azabicyclo[3.2.0]heptane-2-carboxylic acid 4,4-dioxide | Present | Absent | Absent | Absent | Absent | Absent |
|  | Chloramphenicol | Present | Present | Absent | Absent | Absent | Absent |
|  | Chloramphenicol acetate | Present | Absent | Absent | Absent | Absent | Absent |
|  | D-(−)-ampicillin | Present | Present | Absent | Absent | Absent | Absent |
|  | Dapsone | Present | Absent | Absent | Absent | Absent | Absent |
|  | Pyrimidinethione | Present | Absent | Absent | Absent | Absent | Absent |
|  | Sulfabenzamide | Present | Present | Absent | Absent | Absent | Absent |
|  | Sulfacetamide | Present | Present | Absent | Absent | Absent | Absent |
|  | Sulfanilamide | Present | Present | Absent | Absent | Absent | Absent |
|  | Sulfanilic acid | Present | Present | Absent | Absent | Absent | Absent |
| **Dye intermediate** | Sudan Blue | Present | Present | Absent | Absent | Absent | Absent |
|  | Sudan III | Present | Present | Absent | Absent | Absent | Absent |
|  | 4-mercaptobenzenesulfonic acid | Present | Present | Absent | Absent | Absent | Absent |
|  | 1,3-Bis[4-(diethylamino)phenyl]thiourea | Present | Present | Absent | Absent | Absent | Absent |
|  | 2-Hydroxyethyl (2,4-dichlorophenoxy)acetate | Present | Present | Absent | Absent | Absent | Absent |
|  | 6,8-Dichloro-4-hydroxy-2H-1,4-benzoxazin-3(4H)-one | Present | Present | Absent | Absent | Absent | Absent |
| **Pesticide** | (1-Aminocyclohexyl)acetic acid | Present | Present | Absent | Absent | Absent | Absent |
|  | 1-[2,2,2-Trichloro-1-(2-methylphenyl)ethyl]naphthalene | Present | Present | Absent | Absent | Absent | Absent |
|  | Emamectin B1a | Present | Present | Absent | Absent | Absent | Absent |
|  | O-Methyl S-phenyl methylphosphonodithioate | Present | Present | Absent | Absent | Absent | Absent |
|  | Thiamethoxam | Present | Present | Absent | Absent | Absent | Absent |
|  | 2-[2-(Tetradecyloxy)ethoxy]ethyl hydrogen sulfate | Present | Present | Absent | Absent | Absent | Absent |
|  | 3-Hydroxy-2,2-bis(hydroxymethyl)propyl myristate | Present | Present | Absent | Absent | Absent | Absent |
|  | 4-Caproylresorcinol | Present | Present | Absent | Absent | Absent | Absent |
|  | Hexadecyl diphenyl phosphate | Present | Present | Absent | Absent | Absent | Absent |
|  | 3-(Trifluoromethyl)phenylacetic acid | Present | Present | Absent | Absent | Absent | Absent |
|  | 3-{[4-([AMINO(IMINO)METHYL]AMINOSULFONYL)ANILINO]METHYLENE}-2-OXO-2,3-DIHYDRO-1H-INDOLE | Present | Present | Absent | Absent | Absent | Absent |
|  | 3-acetamido-4-methyl-2-nitrobenzoic acid | Present | Present | Absent | Absent | Absent | Absent |
|  | 3-Heptyl-6,7-dimethyl-7H-[1,2,4]triazolo[3,4-b][1,3,4]thiadiazine | Present | Present | Absent | Absent | Absent | Absent |
|  | 6,8-Dichloro-4-hydroxy-2H-1,1-benzoxazin-3(4H)-one | Absent | Present | Absent | Absent | Absent | Absent |
|  | Desaminometribuzin | Present | Present | Absent | Absent | Absent | Absent |
|  | Metamiton | Present | Present | Absent | Absent | Absent | Absent |
|  | Pyrazosulfuron-ethyl | Present | Present | Absent | Absent | Absent | Absent |
|  | 4-Chloroaniline-2-sulfonic acid | Present | Present | Absent | Absent | Absent | Absent |
|  | Clothianidin | Absent | Present | Absent | Absent | Absent | Absent |
|  | 1-Petyloxy-2,3-difluorobenzene | Present | Present | Absent | Absent | Absent | Absent |
|  | N,N'-Thiocarbonyldiimidazole | Absent | Present | Absent | Absent | Absent | Absent |
|  | Triethyl phosphate | Present | Present | Absent | Absent | Absent | Absent |
|  | Nootkatone | Present | Present | Absent | Absent | Absent | Absent |
|  | (3beta)-Cholest-5-en-3-yl methylcarbamate | Present | Present | Absent | Absent | Present | Absent |
|  | 1-(Dichloromethyl)urea | Present | Present | Absent | Absent | Present | Absent |
|  | 3,5,6-Trichloro-2-pyridinol | Present | Present | Absent | Absent | Present | Present |
|  | Dursban | Present | Present | Absent | Absent | Present | Present |
|  | Malathion | Present | Present | Absent | Absent | Present | Present |
|  | Diazinon | Present | Present | Absent | Absent | Present | Present |
|  | Parathion | Present | Present | Present | Absent | Present | Present |
|  | Dimethoate | Present | Present | Present | Absent | Present | Present |
|  | Methamidophos | Present | Present | Present | Present | Present | Present |
|  | Tebufenozide | Present | Present | Present | Present | Present | Present |
|  | Imidacloprid | Present | Present | Present | Present | Present | Present |
|  | Bifenthrin | Present | Present | Present | Present | Present | Present |
|  | Beta-cyfluthrin | Present | Present | Present | Present | Present | Present |
|  | Avermectin | Present | Present | Present | Present | Present | Present |
|  | 2,4-Dichlorophenoxyacetic acid | Present | Present | Present | Present | Present | Present |
|  | N,N'-Dimethyl-4,4'-bipyridinium dichloride | Present | Present | Present | Present | Present | Present |
|  | (RS)-5-amino-1-(2,6-dichloro-4-trifluoromethylphenyl)-4-trifluoromethylphenyl)-4-(trifluoromethyl)-1H-pyrazole-3-carbonitrile | Present | Present | Present | Present | Present | Present |
|  | Fipronil Sulfoxide | Present | Present | Present | Present | Present | Present |
|  | 3-(2,6-Dichloro-4-(trifluoromethyl)phenyl)-1-(1,2,4-thiadiazol-3-yl)-2-buten-1-ol | Present | Present | Present | Present | Present | Present |
|  | 1,1'-Dimethyl-4,4'-bipyridinium chloride | Present | Present | Present | Present | Present | Present |
|  | Methyl€-2-[2-92-amino-1,3-thiazol-4-yl)-1-benzofuran-3-yl]-3-methoxyacrylate | Present | Present | Present | Present | Present | Present |
|  | 4-bromo-2-phenyl-1,3-benzodioxole-5-carboxamide | Present | Present | Present | Present | Present | Present |
|  | Nitenpyram | Present | Present | Present | Present | Present | Present |
|  | 1-[(6-Chloro-3-pyridinyl)methyl]-N-nitro-2-[(2-guanidinyl)imino]imidazolidinimine | Present | Present | Present | Present | Present | Present |
| **PPCPs** | Lauramidopropyl dimethylamine | Present | Absent | Absent | Absent | Absent | Absent |
|  | pendecamaine | Present | Absent | Absent | Absent | Absent | Absent |
|  | Clofibric acid | Present | Absent | Absent | Absent | Absent | Absent |
|  | 2-(3-Nitrophenoxy)-N-[4-(4-oxo-4H-3,1-benzoxazin-2-yl)phenyl]acetamide | Present | Absent | Absent | Absent | Absent | Absent |
|  | 3-(tert-Butyl)-1,2,4-thiadiazol-5-amine | Present | Absent | Absent | Absent | Absent | Absent |
|  | Thiocarbonyldiimidazole | Present | Absent | Absent | Absent | Absent | Absent |
|  | 4-(3-Isopropyl-1,2,4-oxadiazol-5-yl)piperidine | Present | Absent | Absent | Absent | Absent | Absent |
|  | 4-(4-Aminophenyl)-1H-indazol-3-amine | Present | Absent | Absent | Absent | Absent | Absent |
|  | 4-(Phenylsulfonyl)naphtho[2,3-c]furan-1,3-dione | Present | Present | Absent | Absent | Absent | Absent |
|  | 4-[3-(benzylamino)butyl]-2-methoxyphenol | Present | Present | Absent | Absent | Absent | Absent |
|  | 4-Amino-5,5,5-trifluoropentanoic acid | Present | Present | Absent | Absent | Absent | Absent |
|  | 4-Aminophenol | Present | Present | Absent | Absent | Absent | Absent |
|  | 4-Ethoxybenzenesulfonyl chloride | Present | Present | Absent | Absent | Absent | Absent |
|  | 4-Hydroxy-2-(methylthio)pyrimidine-5-carboxylic acid | Present | Present | Absent | Absent | Absent | Absent |
|  | 4-Methoxybenzenesulfonamide | Present | Present | Absent | Absent | Absent | Absent |
|  | 4-methylpyridine-3-sulfonic acid | Present | Present | Absent | Absent | Absent | Absent |
|  | 4-Nitro-sulfamethoxazole | Present | Present | Absent | Absent | Absent | Absent |
|  | 4-p-Chlorobenzoylphenol | Present | Present | Absent | Absent | Absent | Absent |
|  | 5-bromo-2-(4-chlorophenyl)thiazole-4-carboxylic acid | Present | Present | Absent | Absent | Absent | Absent |
|  | 5-Chloro-2-methoxybenzenesulfonyl chloride | Present | Present | Absent | Absent | Absent | Absent |
|  | 6,9-Dichloro-1,2,3,4-tetrahydroacridine | Present | Present | Absent | Absent | Absent | Absent |
|  | 6-Amino-1,3-dimethyluracil | Present | Present | Absent | Absent | Absent | Absent |
|  | 8-[(tert-Butoxycarbonyl)amino]-1,4-dioxaspiro[4.5]decane-8-carboxylic acid | Present | Present | Absent | Absent | Absent | Absent |
|  | 8-Benzyl-8-azabicyclo(3.2.1)octan-3-one | Present | Present | Absent | Absent | Absent | Absent |
|  | 8-Hydroxy-7-{[hydroxy(4-nitrophenyl)acetyl]amino}-5-quinolinesulfonic acid | Present | Present | Absent | Absent | Absent | Absent |
|  | 9-Hydroxyellipticine | Present | Present | Absent | Absent | Absent | Absent |
|  | Acetylsulfanilamide | Present | Present | Absent | Absent | Absent | Absent |
|  | Amifloxacin | Present | Present | Absent | Absent | Absent | Absent |
|  | Amrinone | Present | Present | Absent | Absent | Absent | Absent |
|  | Avermectin B1a | Present | Present | Absent | Absent | Absent | Absent |
|  | Benzylacyclouridine | Present | Present | Absent | Absent | Absent | Absent |
|  | Chlorosulfamic acid | Present | Present | Absent | Absent | Absent | Absent |
|  | Diclofenac | Present | Present | Absent | Absent | Absent | Absent |
|  | diphenyl-1,3,4-oxadiazole | Present | Present | Absent | Absent | Absent | Absent |
|  | Dobutamine | Present | Present | Absent | Absent | Absent | Absent |
|  | ethyl 6-acetyl-2-amino-4H,5H,6H,7H-thieno[2,3-c]pyridine-3-carboxylate | Present | Present | Absent | Absent | Absent | Absent |
|  | Medifoxamine | Present | Present | Absent | Absent | Absent | Absent |
|  | methaqualone | Present | Present | Absent | Absent | Absent | Absent |
|  | Methyl (2E)-3,3,3-trifluoro-2-[(phenylsulfonyl)imino]propanoate | Present | Present | Absent | Absent | Absent | Absent |
|  | methylphenylsulfoxide | Present | Present | Absent | Absent | Absent | Absent |
|  | N-benzoyl-N'-(4-fluorophenyl)thiourea | Present | Present | Absent | Absent | Absent | Absent |
|  | Nifurpirinol | Present | Present | Absent | Absent | Absent | Absent |
|  | N-propylnorapomorphine | Present | Present | Absent | Absent | Absent | Absent |
|  | O,O-Diethyl thiophosphate | Present | Present | Absent | Absent | Absent | Absent |
|  | procodazole | Present | Present | Absent | Absent | Absent | Absent |
|  | Propranolol | Present | Present | Absent | Absent | Absent | Absent |
|  | Riluzole | Present | Present | Absent | Absent | Absent | Absent |
|  | Tetracycline | Present | Present | Absent | Absent | Absent | Absent |
|  | Thioxocerium | Present | Present | Absent | Absent | Absent | Absent |
|  | tiopinac | Present | Present | Absent | Absent | Absent | Absent |
|  | Troxacitabine | Present | Present | Absent | Absent | Absent | Absent |
|  | Methyl 4-bromo-3-methoxy-2-thiophenecarboxylate | Present | Present | Absent | Absent | Absent | Absent |
|  | p-Toluenesulfonic acid | Present | Present | Absent | Absent | Absent | Absent |
|  | (2E,4Z)-N-(2-Methylbutyl)-2,4-undecadiene-8,10-diynamide | Present | Present | Absent | Absent | Absent | Absent |
|  | (2S,3R)-3-(2,4-Difluorophenyl)-2-(5-fluoro-4-pyrimidinyl)-3-pentanol | Present | Present | Absent | Absent | Absent | Absent |
|  | [(6-Chloro-2,3-dihydroimidazo[2,1-b][1,3]thiazol-5-yl)methoxy]acetonitrile | Present | Present | Absent | Absent | Absent | Absent |
|  | [1-Cyclohexyl-4-(cyclohexylamino)-5-phenyl-1H-pyrrol-3-yl](phenyl)methanone | Present | Present | Absent | Absent | Absent | Absent |
|  | [3-(3,4-Dichlorophenyl)-2-oxo-1,3-oxazolidin-5-yl]methyl carbamate | Present | Present | Absent | Absent | Absent | Absent |
|  | [3-(4-Ethoxy-3,5-dimethoxyphenyl)-6-phenyl-7H-[1,2,4]triazolo[3,4-b][1,3,4]thiadiazin-7-yl]acetic acid | Present | Present | Absent | Absent | Absent | Absent |
|  | [3-(Ethoxycarbonyl)phenyl]sulfamic acid | Present | Present | Absent | Absent | Absent | Absent |
|  | 1-(1,2,3,6-Tetrahydro-4-pyridinyl)-1,3-dihydro-2H-benzimidazol-2-one | Present | Present | Absent | Absent | Absent | Absent |
|  | 1-(methylthio)-3,4-dihydropyrido[2,1-a]isoindole-2,6-dione | Present | Present | Absent | Absent | Absent | Absent |
|  | 1-(p-nitrophenyl)-2-amino-1,3-propanediol | Present | Present | Absent | Absent | Absent | Absent |
|  | 1,2-Dihydro-1,5-dimethyl-2-phenyl-4-(4-quinazolinylamino)-3H-pyrazol-3-one | Present | Present | Absent | Absent | Absent | Absent |
|  | 1-[(2-Chloro-1,3-thiazol-5-yl)methyl]-3-methylguanidine | Present | Present | Absent | Absent | Absent | Absent |
|  | 10-Methyl-9(10H)-acridone | Present | Present | Absent | Absent | Absent | Absent |
|  | 17-(4-Fluorophenoxy)-3,6,9,12,15-pentaoxaheptadecan-1-ol | Present | Present | Absent | Absent | Absent | Absent |
|  | 1-Boc-3-Formylindole | Present | Present | Absent | Absent | Absent | Absent |
|  | 1-Methyl-4-(2-propoxybenzamido)-3-propyl-1H-pyrazole-5-carboxamide | Present | Present | Absent | Absent | Absent | Absent |
|  | 2-(1H-Imidazol-1-ylmethyl)cyclohexanone oxime | Present | Present | Absent | Absent | Absent | Absent |
|  | 2-(2-Chlorobenzyl)-4,4-dimethyl-5-(pentyloxy)-1,2-oxazolidin-3-one | Present | Present | Absent | Absent | Absent | Absent |
|  | 2-(4-Ethoxy-3,5-dimethoxyphenyl)-6-(2-nitrophenyl)imidazo[2,1-b][1,3,4]thiadiazole | Present | Present | Absent | Absent | Absent | Absent |
|  | 2-(4-Imino-1-methyl-1,4-dihydro-5H-pyrazolo[3,4-d]pyrimidin-5-yl)ethanol | Present | Present | Absent | Absent | Absent | Absent |
|  | 2-(5H-[1,2,4]Triazino[5,6-b]indol-3-ylsulfanyl)acetamide | Present | Present | Absent | Absent | Absent | Absent |
|  | 2-(Cyclohexylmethyl)-4,5-dihydro-1H-imidazole | Present | Present | Absent | Absent | Absent | Absent |
|  | 2-(Diethylamino)ethyl acetate | Present | Present | Absent | Absent | Absent | Absent |
|  | 2-(Methylsulfanyl)-4-phenyl-6-(trifluoromethyl)pyrimidine | Present | Present | Absent | Absent | Absent | Absent |
|  | 2-[(2-Chlorophenoxy)methyl]-7-iodo-3-(2-pyridinyl)-4(3H)-quinazolinone | Present | Present | Absent | Absent | Absent | Absent |
|  | 2-{[4-hydroxy-6-(trifluoromethyl)pyrimidin-2-yl]sulfanyl}acetic acid | Present | Present | Absent | Absent | Absent | Absent |
|  | 2-{[5-Acetyl-3-cyano-6-methyl-4-(3-nitrophenyl)-1,4-dihydro-2-pyridinyl]sulfanyl}acetamide | Present | Present | Absent | Absent | Absent | Absent |
|  | 2-Amino-4-isopropylthiazole | Present | Present | Absent | Absent | Absent | Absent |
|  | 2-Methyl-4-oxo-4-[(4-sulfamoylphenyl)amino]-2-butenoic acid | Present | Present | Absent | Absent | Absent | Absent |
|  | 3-(1-Aminocyclohexyl)-1-propanol | Present | Present | Absent | Absent | Absent | Absent |
|  | 3-(Allylsulfanyl)-4-amino-6-methyl-1,2,4-triazin-5(4H)-one | Present | Present | Absent | Absent | Absent | Absent |
|  | neflumozide | Present | Present | Absent | Absent | Absent | Absent |
|  | (3a'R,5'R,6'S,6a'R)-5'-[(2R)-1,4-Dioxaspiro[4.5]dec-2-yl]-6'-methoxytetrahydrospiro[cyclohexane-1,2'-furo[2,3-d][1,3]dioxole] | Present | Present | Absent | Absent | Absent | Absent |
|  | (1E)-1-Tetradecene-1,14-disulfonic acid | Present | Present | Absent | Absent | Absent | Absent |
|  | Equol | Present | Present | Absent | Absent | Absent | Absent |
|  | Ethyl (dianilinophosphoryl)carbamate | Present | Present | Absent | Absent | Absent | Absent |
|  | [(4-OXO-6-PHENYL-1H-PYRIMIDIN-2-YL)SULFANYL]ACETIC ACID | Present | Present | Absent | Absent | Absent | Absent |
|  | 1,6-Bismaleimidoethane | Present | Present | Absent | Absent | Absent | Absent |
|  | 3'-Amino-2',3'-dideoxyadenosine | Present | Present | Absent | Absent | Absent | Absent |
|  | Bis[2-(diethylamino)ethyl] (methylenedi-4,1-phenylene)biscarbamate | Present | Present | Absent | Absent | Absent | Absent |
|  | N-(1,3,4,5-Tetrahydroxy-2-octadecanyl)hexadecanamide | Present | Present | Absent | Absent | Absent | Absent |
|  | 7H-[1,2,4]Triazolo[4,3-b][1,2,4]triazole-3,7-diamine | Present | Present | Absent | Absent | Absent | Absent |
|  | N-(2-aminoethyl)stearamide | Present | Present | Absent | Absent | Absent | Absent |
|  | N-Boc-2-hydroxyethylpiperidine | Present | Present | Absent | Absent | Absent | Absent |
|  | 1-(4-Imidazol-1-yl-phenyl)-ethanone | Present | Present | Absent | Absent | Absent | Absent |
|  | CORTISONE 21-CYCLOPENTANEPROPIONATE | Present | Present | Absent | Absent | Absent | Absent |
|  | Diethyl phthalate | Present | Present | Absent | Absent | Absent | Absent |
|  | 2-Sulfamoylacetamide | Present | Present | Absent | Absent | Absent | Absent |
|  | 4-Amino-1,2,5-oxadiazole-3-carboxamide 2-oxide | Present | Present | Absent | Absent | Absent | Absent |
|  | Ethyl [(5-amino-1H-1,2,4-triazol-3-yl)sulfanyl]acetate | Present | Present | Absent | Absent | Absent | Absent |
|  | gamma-Acetylenic gaba | Present | Present | Absent | Absent | Absent | Absent |
|  | N-(2-Aminoethyl)-N-(2-hydroxyethyl)dodecanamide | Present | Present | Absent | Absent | Absent | Absent |
|  | N-Boc-piperidine-3-methanol | Present | Present | Absent | Absent | Absent | Absent |
|  | 2,6-Bis(bromomethyl)piperidine | Present | Present | Absent | Absent | Absent | Absent |
|  | Butyrophenone | Present | Present | Absent | Absent | Absent | Absent |
|  | 1-aminobenzimidazole-2-sulfonic acid | Present | Present | Absent | Absent | Absent | Absent |
|  | 4-(Methoxymethyl)-4-phenyl-1-(3,3,3-triphenylpropyl)piperidine | Present | Present | Absent | Absent | Absent | Absent |
|  | 5-(methylthio)-1,3,4-thiadiazol-2-amine | Present | Present | Absent | Absent | Absent | Absent |
|  | Azelaic acid | Present | Present | Absent | Absent | Absent | Absent |
|  | Ethosuximide | Present | Present | Absent | Absent | Absent | Absent |
|  | memantine | Present | Present | Absent | Absent | Absent | Absent |
|  | N-(2-Hydroxyethyl)-N-tetradecyloctadecanamide | Present | Present | Absent | Absent | Absent | Absent |
|  | N-(2-hydroxyethyl)eicosa-5,8,11,14-tetraenamide | Present | Present | Absent | Absent | Absent | Absent |
|  | N,N'-(1R,2R)-1,2-Cyclohexanediyldioctadecanamide | Present | Present | Absent | Absent | Absent | Absent |
|  | piroximone | Present | Present | Absent | Absent | Absent | Absent |
|  | 2,4-Dichloro-6-(pentabromophenoxy)-1,3,5-triazine | Present | Present | Absent | Absent | Absent | Absent |
|  | 2,2-Difluorocyclopropanecarbaldehyde | Present | Present | Absent | Absent | Absent | Absent |
|  | N-(4,6-Dimethyl-2-pyrimidinyl)-N-ethyl-1,4-benzenediamine | Present | Present | Absent | Absent | Absent | Absent |
|  | N-[(3S,4R,7R,8R,9S)-7-Butyl-8-hydroxy-4,9-dimethyl-2,6-dioxo-1,5-dioxonan-3-yl]-3-formamido-2-hydroxybenzamide | Present | Present | Absent | Absent | Absent | Absent |
|  | 1-[2,2-Bis(2-ethylhexyl)hydrazino]-4-methyl-7,7a-dihydro-1H-benzotriazole | Present | Present | Absent | Absent | Absent | Absent |
|  | Myristamidopropyl betaine | Present | Present | Absent | Absent | Absent | Absent |
|  | 1-(2-carboxylatoethyl)-2-(heptadec-8-enyl)-4,5-dihydro-1-(2-hydroxyethyl)-1H-imidazolium | Present | Present | Absent | Absent | Absent | Absent |
|  | 1,2,3-Benzotriazole | Present | Present | Absent | Absent | Absent | Absent |
|  | 1,3-Dimethyl-1,7a-dihydro-7H-pyrazolo[4,3-d]pyrimidin-7-one | Present | Present | Absent | Absent | Absent | Absent |
|  | 15-Anilinoretinal | Present | Present | Absent | Absent | Absent | Absent |
|  | 1-phenethylpiperidine | Present | Present | Absent | Absent | Absent | Absent |
|  | 2,5-Diamino-1,4-benzenedisulfonic acid | Present | Present | Absent | Absent | Absent | Absent |
|  | 2,6-Di-tert-butylanthracene | Present | Present | Absent | Absent | Absent | Absent |
|  | 2-heptadecyl-4-methyl-2-oxazoline-4-methanol | Present | Present | Absent | Absent | Absent | Absent |
|  | 2-Selenophenecarbaldehyde | Present | Present | Present | Absent | Absent | Absent |
|  | 3,5-DIMETHYLPYRAZOL-1-METHANOL | Present | Present | Present | Absent | Absent | Absent |
|  | 3-(3-Cyanopropyl)-1-methyl-1H-imidazolium | Present | Present | Present | Absent | Absent | Absent |
|  | 4-Ethyl-4,5-dihydro-2-undecyl-4-oxazolemethanol | Present | Present | Present | Absent | Absent | Absent |
|  | 4-[(4,6-Dichloropyrimidin-2-yl)amino]benzonitrile | Present | Present | Present | Absent | Absent | Absent |
|  | 4-Cyclohexyl-2-(2-cyclohexylethyl)-N-ethylbutanamide | Present | Present | Present | Absent | Absent | Absent |
|  | 5-[[(7-Chloro-4-quinolinyl)thio]methyl]-2,4-dihydro-4-methyl-3H-1,2,4-triazole-3-thione | Present | Present | Present | Absent | Absent | Absent |
|  | Betaine | Present | Present | Present | Absent | Absent | Absent |
|  | Methylphenobarbital | Present | Present | Present | Absent | Absent | Absent |
|  | Myristamine oxide | Present | Present | Present | Absent | Absent | Absent |
|  | methyprylon | Present | Present | Present | Absent | Absent | Absent |
|  | N-(2-Hydroxypropyl)-3,5,5-trimethylhexanamide | Present | Present | Present | Absent | Absent | Absent |
|  | N-(hydroxymethyl)stearamide | Present | Present | Present | Absent | Absent | Absent |
|  | N-[(2S,3R)-1,3-Dihydroxy-2-octadecanyl]butanamide | Present | Present | Present | Absent | Absent | Absent |
|  | (3,5-Bis{2-[2-(2-methoxyethoxy)ethoxy]ethoxy}phenyl)methanol | Present | Present | Present | Absent | Absent | Absent |
|  | Inspra | Present | Present | Present | Absent | Absent | Absent |
|  | spisulosine | Present | Present | Present | Absent | Absent | Absent |
|  | 5-Allyl-1H-imidazole | Present | Present | Present | Absent | Absent | Absent |
|  | 5-Pentyl-1,3,4-thiadiazol-2-amine | Present | Present | Present | Absent | Absent | Absent |
|  | Amdoxovir | Present | Present | Present | Absent | Absent | Absent |
|  | 1H,3H-[1,2,5]Thiadiazolo[3,4-c][1,2,5]thiadiazole | Present | Present | Present | Absent | Absent | Absent |
|  | 1-Tosyl-1H-imidazole-4-carboxylic acid | Present | Present | Present | Absent | Absent | Absent |
|  | 2-(chloroacetyl)-6,7-dimethoxy-1,2,3,4-tetrahydroisoquinoline | Present | Present | Present | Absent | Absent | Absent |
|  | 3-Methyl-6-(trifluoromethyl)[1,2]oxazolo[3,4-b]pyridin-4(1H)-one | Present | Present | Present | Absent | Absent | Absent |
|  | 5-(2,4,5-Trichlorophenyl)-1,2-dihydro-3H-1,2,4-triazol-3-one | Present | Present | Present | Absent | Absent | Absent |
|  | 5-Amino-1H-pyrazole-1-ethanol | Present | Present | Present | Absent | Absent | Absent |
|  | C14-Dihydroceramide | Present | Present | Present | Absent | Absent | Absent |
|  | Hexadecanamide | Present | Present | Present | Absent | Absent | Absent |
|  | Myristamidopropyl dimethylamine | Present | Present | Present | Absent | Absent | Absent |
|  | N,N-Bis(2-ethylhexyl)octanamide | Present | Present | Present | Absent | Absent | Absent |
|  | tert-Butyl 4-((tert-butoxycarbonyl)amino)-4-methylpiperidine-1-carboxylate | Present | Present | Present | Absent | Absent | Absent |
|  | tert-Butyl 4-(3-chloropropyl)piperazine-1-carboxylate | Present | Present | Present | Absent | Absent | Absent |
|  | tert-butyl 4-(3-ethoxy-3-oxopropyl)piperidine-1-carboxylate | Present | Present | Present | Absent | Absent | Absent |
|  | Linoleamide | Present | Present | Present | Absent | Absent | Absent |
|  | Capsi-amide | Present | Present | Present | Absent | Absent | Absent |
|  | N-(4-{Bis[4-(diethylamino)-2-methylphenyl]methyl}-2-methoxyphenyl)octanamide | Present | Present | Present | Absent | Absent | Absent |
|  | 2-[2-(2-Chloro-1,1,2-trifluoroethoxy)phenyl]-5-[[4-(hexadecylsulfonyl)phenyl]amino]-2,4-dihydro-3H-pyrazol-3-one | Present | Present | Present | Absent | Absent | Absent |
|  | 2-{2-Hydroxy-3-[(2-methyl-2-propanyl)amino]propoxy}-N-(5-methylhexyl)-1,3-thiazole-5-carboxamide | Present | Present | Present | Absent | Absent | Absent |
|  | 4-(4-Methoxyphenyl)-1H-1,2,3-triazole-5-carbonitrile | Present | Present | Present | Absent | Absent | Absent |
|  | 4-[4,6-Di(biphenyl-4-yl)-1,3,5-triazin-2-yl]benzene-1,3-diol | Present | Present | Present | Absent | Absent | Absent |
|  | butoctamide | Present | Present | Present | Absent | Absent | Absent |
|  | Docosanamide | Present | Present | Present | Absent | Present | Absent |
|  | ethyl 5-tert-butyl-1H-pyrazole-3-carboxylate | Present | Present | Present | Absent | Present | Absent |
|  | N-Methyldodecanamide | Present | Present | Present | Absent | Present | Absent |
|  | N,N′-Methylenebisacrylamide | Present | Present | Present | Absent | Present | Absent |
|  | Octadecylacrylamide | Present | Present | Present | Absent | Present | Absent |
|  | Tricosanamid | Present | Present | Present | Absent | Present | Absent |
|  | N-(3-Aminopropyl)octadecanamide | Present | Present | Present | Absent | Present | Absent |
|  | N-[1-(Difluoroamino)cyclohexyl]-N,2-difluoroacetamide | Present | Present | Present | Absent | Present | Absent |
|  | Lignoceroyl Ethanolamide | Present | Present | Present | Absent | Present | Absent |
|  | CERAMIDE AP | Present | Present | Present | Absent | Present | Absent |
|  | (12-Crown-4)-2-methanol | Present | Present | Present | Absent | Present | Absent |
|  | 2-(8-Heptadecen-1-yl)-4,5-dihydro-1,3-oxazole | Present | Present | Present | Absent | Present | Absent |
|  | 2,4,6-quinazolinetriamine | Present | Present | Present | Absent | Present | Absent |
|  | 4,4-Difluororetinoic acid | Present | Present | Present | Absent | Present | Absent |
|  | 6-isothiocyanatohexylbenzene | Present | Present | Present | Absent | Present | Absent |
|  | Chlorodimethoxyborane | Present | Present | Present | Absent | Present | Absent |
|  | Ethyl 2-{[1-(4-chlorophenyl)-1H-1,2,4-triazol-3-yl]oxy}propanoate | Present | Present | Present | Absent | Present | Absent |
|  | ibufenac | Present | Present | Present | Present | Present | Absent |
|  | METHYL 1,4-DI-BOC-PIPERAZINE-2-ACETATE | Present | Present | Present | Present | Present | Absent |
|  | Myristamide | Present | Present | Present | Present | Present | Present |
|  | N,N-Diethyl-2,4-dimethylbenzamide | Present | Present | Present | Present | Present | Present |
|  | N,N-Dimethyloleamide | Present | Present | Present | Present | Present | Present |
|  | N,N'-1,6-Hexanediylbis(2-ethylhexanamide) | Present | Present | Present | Present | Present | Present |
|  | N,N'-Bis[(4-methyl-1-piperazinyl)methyl]succinamide | Present | Present | Present | Present | Present | Present |
|  | PKI166 | Present | Present | Present | Present | Present | Present |
|  | Vigabatrin | Present | Present | Present | Present | Present | Present |
|  | Vildagliptin | Present | Present | Present | Present | Present | Present |
|  | N-(3,5-Dichloro-2-pyridinyl)-2-fluoroacetamide | Present | Present | Present | Present | Present | Present |
|  | N-(4-(Cyclohexylmethyl)cyclohexyl)acetamide | Present | Present | Present | Present | Present | Present |
|  | 1-Boc-piperidine-4-carboxaldehyde | Present | Present | Present | Present | Present | Present |
|  | (17alpha)-17-{2-[Ethyl(phenyl)amino]-1,3-thiazol-4-yl}-17-hydroxyandrost-4-en-3-one | Present | Present | Present | Present | Present | Present |
|  | (1E)-2-(1-Naphthyl)-N-{2-(1-naphthyl)-2-[2-(1-piperidinyl)ethyl]hexyl}-2-[2-(1-piperidinyl)ethyl]-1-hexanimine | Present | Present | Present | Present | Present | Present |
|  | [(6-Heptadecyl-1,3,5-triazine-2,4-diyl)diimino]dimethanol | Present | Present | Present | Present | Present | Present |
|  | {2-[(8E)-8-Heptadecen-1-yl]-4,5-dihydro-1,3-oxazole-4,4-diyl}dimethanol | Present | Present | Present | Present | Present | Present |
|  | 1,1'-(Phenylphosphinediyl)dipiperidine | Present | Present | Present | Present | Present | Present |
|  | 1,5-Dithiaspiro[5.11]heptadec-7-ylmethanol | Present | Present | Present | Present | Present | Present |
|  | 1-Boc-4-Vinylpiperidine | Present | Present | Present | Present | Present | Present |
|  | 1-Phenyl-5-(1H-pyrrol-1-yl)-1H-pyrazole-4-carbohydrazide | Present | Present | Present | Present | Present | Present |
|  | 2-(1H-Benzotriazol-1-ylacetyl)-N-phenylhydrazinecarbothioamide | Present | Present | Present | Present | Present | Present |
|  | 2-(2-tert-Butyl-1,3-thiazol-4-yl)acetohydrazide | Present | Present | Present | Present | Present | Present |
|  | 2-(Dimethylamino)-N,N'-bis(2-methyl-2-propanyl)hexanediamide | Present | Present | Present | Present | Present | Present |
|  | 2,2,2-Trifluoroacetamide | Present | Present | Present | Present | Present | Present |
|  | 2,2-Dichloro-N-(2-propyn-1-yl)acetamide | Present | Present | Present | Present | Present | Present |
|  | 2,3,5,5-Tetramethyl-4-oxo-1,3-thiazolidine-2-carboxylic acid 1,1-dioxide | Present | Present | Present | Present | Present | Present |
|  | 2,5-Dichlorosulfanilic Acid | Present | Present | Present | Present | Present | Present |
|  | 2,5-di-tert-Butylhydroquinone | Present | Present | Present | Present | Present | Present |
|  | 2-Heptadecylimidazole | Present | Present | Present | Present | Present | Present |
|  | 2-Methyl-2-propanyl 4,4'-bipiperidine-1-carboxylate | Present | Present | Present | Present | Present | Present |
|  | 2-Methyl-6-tridecylpiperidine | Present | Present | Present | Present | Present | Present |
|  | 2-Pentadecyl-1H-imidazole | Present | Present | Present | Present | Present | Present |
|  | 2-Piperidinylmethanol | Present | Present | Present | Present | Present | Present |
|  | 3-(2,6-dichlorophenyl)-5-methylisoxazole-4-carbohydrazide | Present | Present | Present | Present | Present | Present |
|  | 3H-[1,2,5]Oxadiazolo[3,4-c][1,2,6]thiadiazin-7-amine 5,5-dioxide | Present | Present | Present | Present | Present | Present |
|  | 4-(2-Aminoethyl)-1-tritylimidazole | Present | Present | Present | Present | Present | Present |
|  | 4-(2,4,5-Triphenyl-1H-imidazol-1-yl)pyridine | Present | Present | Present | Present | Present | Present |
|  | 4-(Aminoethyl)-1-N-Boc-piperidine | Present | Present | Present | Present | Present | Present |
|  | 4-[2-(Dipropylamino)ethyl]-1,2-benzenediol | Present | Present | Present | Present | Present | Present |
|  | 5-Bromo-6-(4-bromophenyl)-2-(3,4,5-trimethoxyphenyl)imidazo[2,1-b][1,3,4]thiadiazole | Present | Present | Present | Present | Present | Present |
|  | 5,5-Diisopropyl-2-methoxy-3-methyl-1,3-oxazolidin-4-one | Present | Present | Present | Present | Present | Present |
|  | 6-Hydroxy[1,3]thiazolo[4,5-d]pyrimidine-5,7(4H,6H)-dione | Present | Present | Present | Present | Present | Present |
|  | 8-Methyl-3-phenyl-8-azabicyclo[3.2.1]octan-3-ol | Present | Present | Present | Present | Present | Present |
|  | apronalide | Present | Present | Present | Present | Present | Present |
|  | BEHENOYL DIETHANOLAMIDE | Present | Present | Present | Present | Present | Present |
|  | C10-Ceramide | Present | Present | Present | Present | Present | Present |
|  | C16-Dihydroceramide | Present | Present | Present | Present | Present | Present |
|  | CAPRIC DIETHANOLAMIDE | Present | Present | Present | Present | Present | Present |
|  | Carbamazepine | Present | Present | Present | Present | Present | Present |
|  | CERAMIDE 3 | Present | Present | Present | Present | Present | Present |
|  | Cetrimonium | Present | Present | Present | Present | Present | Present |
|  | Dapabutan | Present | Present | Present | Present | Present | Present |
|  | Dichloroacetic acid | Present | Present | Present | Present | Present | Present |
|  | DIHYDROCERAMIDE C8 | Present | Present | Present | Present | Present | Present |
|  | Docosaenoyl Ethanolamide | Present | Present | Present | Present | Present | Present |
|  | Dodecylethanol amide | Present | Present | Present | Present | Present | Present |
|  | Erucamide | Present | Present | Present | Present | Present | Present |
|  | etaqualone | Present | Present | Present | Present | Present | Present |
|  | Ethylhexyl triazone | Present | Present | Present | Present | Present | Present |
|  | Gemfibrozil | Present | Present | Present | Present | Present | Present |
|  | Histamine | Present | Present | Present | Present | Present | Present |
|  | Ketocainol | Present | Present | Present | Present | Present | Present |
|  | Methyl 3-(5-formyl-2-furyl)-2-thiophenecarboxylate | Present | Present | Present | Present | Present | Present |
|  | Methyl 4-Boc-piperazine-2-acetate | Present | Present | Present | Present | Present | Present |
|  | mefexamide | Present | Present | Present | Present | Present | Present |
|  | Myristoleic acid | Present | Present | Present | Present | Present | Present |
|  | Myristyl sulfate | Present | Present | Present | Present | Present | Present |
|  | N-Dodecylacrylamide | Present | Present | Present | Present | Present | Present |
|  | n-octadecylmaleimide | Present | Present | Present | Present | Present | Present |
|  | N,N-Bis(2-hydroxyethyl)dodecanamide | Present | Present | Present | Present | Present | Present |
|  | Palmitoyl ethanolamide | Present | Present | Present | Present | Present | Present |
|  | Padimate A | Present | Present | Present | Present | Present | Present |
|  | PALGLY | Present | Present | Present | Present | Present | Present |
|  | periplocin | Present | Present | Present | Present | Present | Present |
|  | N,N-dimethylsulfamide | Present | Present | Present | Present | Present | Present |
|  | Phenobarbital | Present | Present | Present | Present | Present | Present |
|  | Phenethylamine | Present | Present | Present | Present | Present | Present |
|  | N''-Isopropyl-N'''-{4-[(6-methoxy-1,2,3,4-tetrahydro-8-quinolinyl)amino]pentyl}imidodicarbonimidic diamide | Present | Present | Present | Present | Present | Present |
|  | N~5~-(Diaminomethylene)-N~2~-[(4-methylphenyl)sulfonyl]-N-(4-nitrophenyl)-L-ornithinamide | Present | Present | Present | Present | Present | Present |
|  | N~7~,3-Dibutyl-3H-[1,2,3]triazolo[4,5-d]pyrimidine-5,7-diamine | Present | Present | Present | Present | Present | Present |
|  | Nipam | Present | Present | Present | Present | Present | Present |
|  | Pilocarpine | Present | Present | Present | Present | Present | Present |
|  | Procaine | Present | Present | Present | Present | Present | Present |
|  | Ricinoamidopropyl dimethylamine | Present | Present | Present | Present | Present | Present |
|  | Safingol | Present | Present | Present | Present | Present | Present |
|  | tert-butyl 4-(cyclopropylmethyl)piperidine-1-carboxylate | Present | Present | Present | Present | Present | Present |
|  | tert-Butyl 4-[methoxy(methyl)carbamoyl]piperidine-1-carboxylate | Present | Present | Present | Present | Present | Present |
|  | Dithiocarbamate | Present | Present | Present | Present | Present | Present |
|  | valdipromide | Present | Present | Present | Present | Present | Present |
|  | N-(2,3-Dihydroxypropyl)octadecanamide | Present | Present | Present | Present | Present | Present |
|  | N-(2-Hydroxyhexadecyl)diethanolamine | Present | Present | Present | Present | Present | Present |
|  | N-[(4E)-1,3-Dihydroxy-4-octadecen-2-yl]dodecanamide | Present | Present | Present | Present | Present | Present |
|  | N-[1-Hydroxy-2-(hydroxymethyl)-2-butanyl]-9-octadecenamide | Present | Present | Present | Present | Present | Present |
|  | N-[2-(2-Pentadecyl-4,5-dihydro-1H-imidazol-1-yl)ethyl]hexadecanamide | Present | Present | Present | Present | Present | Present |
|  | N-[2-(Butyrylamino)ethyl]tetradecanamide | Present | Present | Present | Present | Present | Present |
|  | N-[2-(Dimethylamino)ethyl]-N,N',N',N'',N''-pentamethylphosphoric triamide | Present | Present | Present | Present | Present | Present |
|  | N-[3-(4-Morpholinyl)propyl]-10-undecenamide | Present | Present | Present | Present | Present | Present |
|  | N-[3-(Dimethylnitroryl)propyl]octadecanamide | Present | Present | Present | Present | Present | Present |
|  | N-[4-({[6-(2,6-Dichlorophenoxy)-3-pyridinyl]amino}sulfonyl)phenyl]acetamide | Present | Present | Present | Present | Present | Present |
|  | 1-Boc-4-(5-aminopentyl)piperazine | Present | Present | Present | Present | Present | Present |
|  | Arachidoyl Ethanolamide | Present | Present | Present | Present | Present | Present |
|  | N,N'-1,2-Ethanediylbis[N-(3-ethoxypropyl)-10-undecenamide] | Present | Present | Present | Present | Present | Present |
|  | nordoxepin | Present | Present | Present | Present | Present | Present |
|  | N-[3-(5-Methyl-1,3-benzoxazol-2-yl)phenyl]-1-naphthamide | Present | Present | Present | Present | Present | Present |
|  | 2-Hydrazinylthiazole | Present | Present | Present | Present | Present | Present |
|  | 3-(2-Amino-1,3-thiazol-4-yl)benzenesulfonyl fluoride | Present | Present | Present | Present | Present | Present |
|  | Damascenone | Present | Present | Present | Present | Present | Present |
|  | Decanamide | Present | Present | Present | Present | Present | Present |
|  | Hexamine | Present | Present | Present | Present | Present | Present |
|  | Lauramide | Present | Present | Present | Present | Present | Present |
|  | tetrahydroquinoxaline | Present | Present | Present | Present | Present | Present |
|  | [3,3'-bi-1H-1,2,4-triazole]-5,5'-diamine | Present | Present | Present | Present | Present | Present |
|  | N-(1-Hydroxy-2-hexadecanyl)pentadecanamide | Present | Present | Present | Present | Present | Present |
|  | N-(2-Hydroxypropyl)-N-methyldodecanamide | Present | Present | Present | Present | Present | Present |
| **Chemical intermediate** | (Fluoromethoxy)(dimethyl)silane | Present | Present | Absent | Absent | Absent | Absent |
|  | [2-Fluoro-5-(trifluoromethyl)phenyl](phenyl)methanone | Present | Present | Absent | Absent | Absent | Absent |
|  | 1,1'-(2-Butyne-1,4-diyldisulfanediyl)bis(2-methylbenzene) | Present | Present | Absent | Absent | Absent | Absent |
|  | 1,1'-(Adamantan-1-ylphosphoryl)diaziridine | Present | Present | Absent | Absent | Absent | Absent |
|  | 1,1,2,2-Tetraethyldiphosphane 1,2-disulfide | Present | Present | Absent | Absent | Absent | Absent |
|  | 1,2-Diethynyl-3,4,5,6-tetrafluorobenzene | Present | Present | Absent | Absent | Absent | Absent |
|  | 1-[(3xi,9S)-6'-Methoxycinchonan-9-yl]-4-[(9S)-6'-methoxycinchonan-9-yl]-9,10-anthraquinone | Present | Present | Absent | Absent | Absent | Absent |
|  | 2,2'-(1,2-Ethanediyl)ditetrahydrothiophene | Present | Present | Absent | Absent | Absent | Absent |
|  | 2-Acetylbutyrolactone-3,3,4,4-d4 | Present | Present | Absent | Absent | Absent | Absent |
|  | 2-Fluoro-1,3,5-benzenetricarbonitrile | Present | Present | Absent | Absent | Absent | Absent |
|  | 3-(Decyloxy)tetrahydrothiophene 1,1-dioxide | Present | Present | Absent | Absent | Absent | Absent |
|  | 3,7-Dibutyl-3,7-diazabicyclo[3.3.1]nonan-9-one | Present | Present | Absent | Absent | Absent | Absent |
|  | 3,8-Dimethyl-1,2,3,4-tetrahydrodibenzo[b,d]thiophene | Present | Present | Absent | Absent | Absent | Absent |
|  | 3-Methyl-1-[(octylsulfanyl)methyl]-1H-imidazol-3-ium | Present | Present | Absent | Absent | Absent | Absent |
|  | 3-Octyl-2-oxiraneoctanamide | Present | Present | Absent | Absent | Absent | Absent |
|  | 4-(Carboxymethyl)-3-isopropyl-5-oxo-2,5-dihydro-1,2,3-oxadiazol-3-ium | Present | Present | Absent | Absent | Absent | Absent |
|  | 4,4'-[(1,3-Dioxo-1,3-disiloxanediyl)di-2,1-ethanediyl]dibenzenesulfonic acid | Present | Present | Absent | Absent | Absent | Absent |
|  | 4-[(Trifluoromethyl)thio]phenol | Present | Present | Absent | Absent | Absent | Absent |
|  | 4-{[(6E,8E,10E,13E)-1-Carboxy-4-hydroxy-6,8,10,13-nonadecatetraen-5-yl]sulfanyl}benzoic acid | Present | Present | Absent | Absent | Absent | Absent |
|  | 5-Nitro-1,2,3,4-thiatriazole | Present | Present | Absent | Absent | Absent | Absent |
|  | 6,6,6-Trifluoro-1-hexanol | Present | Present | Absent | Absent | Absent | Absent |
|  | 6,6-Bis(difluoroamino)-3-heptanone | Present | Present | Absent | Absent | Absent | Absent |
|  | 6-Quinolinesulfonic acid | Present | Present | Absent | Absent | Absent | Absent |
|  | 9-Acetoanthracene | Present | Present | Absent | Absent | Absent | Absent |
|  | Aza-18-crown-6 | Present | Present | Absent | Absent | Absent | Absent |
|  | Di-9H-fluoren-9-ylidenehydrazine | Present | Present | Absent | Absent | Absent | Absent |
|  | Ethyl N,N-dibenzylglycinate | Present | Present | Absent | Absent | Absent | Absent |
|  | N-(4-Oxocyclohexyl)acetamide | Present | Present | Absent | Absent | Absent | Absent |
|  | Para-Dimethylaminobenzaldehyde | Present | Present | Absent | Absent | Absent | Absent |
|  | Nonoxynol-11 | Present | Present | Absent | Absent | Absent | Absent |
|  | 1,4-Dihydroxy-2-((2-hydroxyethyl)thio)anthraquinone | Present | Present | Absent | Absent | Absent | Absent |
|  | 2-Amino-4-sulfophenol | Present | Present | Absent | Absent | Absent | Absent |
|  | 2-Chloro-4-Aminotoluene-5-Sulfonic Acid | Present | Present | Absent | Absent | Absent | Absent |
|  | Trimethyl pyrophosphate | Present | Present | Absent | Absent | Absent | Absent |
|  | Dimethyl phosphate | Present | Present | Absent | Absent | Absent | Absent |
|  | Tetraethyl pyrophosphate | Present | Present | Absent | Absent | Absent | Absent |
|  | Methylsulfonylmethane | Present | Present | Absent | Absent | Absent | Absent |
|  | Thiacetic acid | Present | Present | Absent | Absent | Absent | Absent |
|  | 2-mercaptoethanol | Present | Present | Absent | Absent | Absent | Absent |
|  | Dodecylbenzenesulfonic acid | Present | Present | Absent | Absent | Absent | Absent |
|  | Nonylphenol ethoxylates | Present | Present | Absent | Absent | Absent | Absent |
|  | Cocmidopropyl betaine | Present | Present | Absent | Absent | Absent | Absent |
|  | Ethoxylated propanol | Present | Present | Absent | Absent | Absent | Absent |
|  | 1,3-Dichloro sulfosuccinate | Present | Present | Absent | Absent | Absent | Absent |
|  | 1,2-ethyl-sodium benzoate | Present | Present | Absent | Absent | Absent | Absent |
|  | Dioctyl Phtalate | Present | Present | Absent | Absent | Absent | Absent |
|  | Acetyl Tributyl Citrate | Present | Present | Absent | Absent | Absent | Absent |
|  | Octyl acetate | Present | Present | Absent | Absent | Absent | Absent |
|  | Celestolide | Present | Present | Absent | Absent | Absent | Absent |
|  | 1,1,3,3-Tetrabromobisphenol A | Present | Present | Absent | Absent | Absent | Absent |
|  | Decabromodiphenyl methanol | Present | Present | Absent | Absent | Absent | Absent |
|  | Hexabromocyclododacane | Present | Present | Absent | Absent | Absent | Absent |
|  | Tris(1,3-dichloro-2-propyl) phosphate | Present | Present | Absent | Absent | Absent | Absent |
|  | Resorcinol bis(2,2-diphenyl phosphate) | Present | Present | Absent | Absent | Absent | Absent |
|  | Perfluorooctane sulfonic acid | Present | Present | Absent | Absent | Absent | Absent |
|  | Perfluorooctanoic acid | Present | Present | Absent | Absent | Absent | Absent |
|  | Perfluorohexanoic acid | Present | Present | Absent | Absent | Absent | Absent |
|  | Perfluorobutanesulfoinc acid | Present | Present | Absent | Absent | Absent | Absent |
|  | Perfluorinated phosphonic acids | Present | Present | Absent | Absent | Absent | Absent |
|  | Perfluoro-1-octanesulfonyl fluoride | Present | Present | Absent | Absent | Absent | Absent |
|  | Hexafluoropropylene oxanoic acid | Present | Present | Absent | Absent | Absent | Absent |
|  | Nylon 6,6-(hexamethylene)diamine | Present | Present | Absent | Absent | Absent | Absent |
|  | Bisphenol A polycarbonate | Present | Present | Absent | Absent | Absent | Absent |
|  | Benzoyl peroxide | Present | Present | Absent | Absent | Absent | Absent |
|  | N-methyl-3-phenyl-3-(4-fluorophenyl)propan-1-amine | Present | Present | Absent | Absent | Absent | Absent |
|  | (RS)-2-(4-(2-methylpropyl)phenyl)propanoic acid | Present | Present | Absent | Absent | Absent | Absent |
|  | 2-chloro-4-ethylamino-6-isopropylamino-s-triazine | Present | Present | Absent | Absent | Absent | Absent |
|  | 5H-dibenzo[b,f]azepine-5-carboxamide | Present | Present | Absent | Absent | Absent | Absent |
|  | (Dimethylamino)-1,4,4a,5,6,11b-hexahydro-3,10,11,12,12a-pentahydroxy-6-methyl-1,11b-dioxo-2-napthacenecarboxamide | Present | Present | Absent | Absent | Absent | Absent |
|  | Hexabromocyclododecane | Present | Present | Absent | Absent | Absent | Absent |
|  | Acesulfame K | Present | Present | Absent | Absent | Absent | Absent |
|  | (-)-Podophyllotoxin | Present | Present | Absent | Absent | Absent | Absent |
|  | (17beta)-4-Iodo-3-oxoandrostan-17-yl 4-methylnicotinate | Present | Present | Absent | Absent | Absent | Absent |
|  | (1E)-3-Bromo-1-iodo-1-propene | Present | Present | Absent | Absent | Absent | Absent |
|  | (1'R,2S,4'S,5S,6R,8'R,10'E,12'S,13'S,14'E,16'E,20'R,21'R,24'S)-21',24'-Dihydroxy-6-isopropyl-5,11',13',22'-tetramethyl-2'-oxo-5,6-dihydrospiro[pyran-2,6'-[3,7,19]trioxatetracyclo[15.6.1.1~4,8~.0~20,24 ~]pentacosa[10,14,16,22]tetraen]-12'-yl 2,6-dideoxy-3-O-methyl-4-O-[2,4,6-trideoxy-3-O-methyl-4-(methylamino)-alpha-L-lyxo-hexopyranosyl]-alpha-L-arabino-hexopyranoside | Present | Present | Absent | Absent | Absent | Absent |
|  | (1Z)-2,2,2-Trifluoro-N-{[(4-methylphenyl)sulfonyl]oxy}-1-phenylethanimine | Present | Present | Absent | Absent | Absent | Absent |
|  | (2E)-3-[4-(Benzyloxy)phenyl]-2-phenylacrylic acid | Present | Present | Absent | Absent | Absent | Absent |
|  | (2E)-3-Phosphonoacrylic acid | Present | Present | Absent | Absent | Absent | Absent |
|  | (2Z)-6-Hydroxy-2-[(2E)-3-phenyl-2-propen-1-ylidene]-1-benzofuran-3(2H)-one | Present | Present | Absent | Absent | Absent | Absent |
|  | (9Z)-N,N-Dibutyl-9-octadecenamide | Present | Present | Absent | Absent | Absent | Absent |
|  | (E)-4-Methoxycinnamic acid | Present | Present | Absent | Absent | Absent | Absent |
|  | (Z)-N,N-dibutyl-1-(octadec-9-enyl)-5-oxopyrrolidine-3-carboxamide | Present | Present | Absent | Absent | Absent | Absent |
|  | 1-(4-Isobutylphenyl)-2,2,4,4-tetramethyl-3-(4-phenyl-1-piperazinyl)cyclobutanol | Present | Present | Absent | Absent | Absent | Absent |
|  | 1-(4-Methoxyphenyl)-3-(4-morpholinyl)-2-propanol | Present | Present | Absent | Absent | Absent | Absent |
|  | 1-(Dodecyloxy)-3-((2-hydroxyethyl)amino)propan-2-ol | Present | Present | Absent | Absent | Absent | Absent |
|  | 1-(Methyloctadecylamino)-2-propanol | Present | Present | Absent | Absent | Absent | Absent |
|  | 1,1'-(Octylimino)dipropan-2-ol | Present | Present | Absent | Absent | Absent | Absent |
|  | 1,1,1,4,4,4-Hexafluoro-2-(trifluoromethyl)-2-butene | Present | Present | Absent | Absent | Absent | Absent |
|  | 1,1,2,2-Tetrafluorodisilanethiol | Present | Present | Absent | Absent | Absent | Absent |
|  | 1,1-Dichloro-2-nitroethene | Present | Present | Absent | Absent | Absent | Absent |
|  | 1,1-Dineopentyloxytrimethylamine | Present | Present | Absent | Absent | Absent | Absent |
|  | 1,6-Hexanediylbis[methyl(undecyl)amine oxide] | Present | Present | Absent | Absent | Absent | Absent |
|  | 1,7-Bis(diisopropylamino)-3,5-diphenyl-3,5-di(2-pyridinyl)-4-heptanone | Present | Present | Absent | Absent | Absent | Absent |
|  | 1,8,15,22,29,36-Hexaazacyclodotetracontane-2,9,16,23,30,37-hexone | Present | Present | Absent | Absent | Absent | Absent |
|  | 1,9,10-Octadecanetriol | Present | Present | Absent | Absent | Absent | Absent |
|  | 10-N-BOC-AMINO-DEC-1-ENE | Present | Present | Absent | Absent | Absent | Absent |
|  | 11-Aminoundecanoic acid | Present | Present | Absent | Absent | Absent | Absent |
|  | 11-Methyl-N-(8-methylnonyl)-1-dodecanamine | Present | Present | Absent | Absent | Absent | Absent |
|  | 12-Aminolauric Acid | Present | Present | Absent | Absent | Absent | Absent |
|  | 12-Hydroxy-N-octadecyloctadecanamide | Present | Present | Absent | Absent | Absent | Absent |
|  | 13-Hydroxyoctadecadienoic acid | Present | Present | Absent | Absent | Absent | Absent |
|  | 18-(Dimethylamino)octadecyl 2-ethylhexanoate | Present | Present | Absent | Absent | Absent | Absent |
|  | 1-Azidopyrene | Present | Present | Absent | Absent | Absent | Absent |
|  | 1-Boc-3-(aminomethyl)piperidine | Present | Present | Absent | Absent | Absent | Absent |
|  | 1-Dodecyl-2-pyrrolidinone | Present | Present | Absent | Absent | Absent | Absent |
|  | (2Z,2'Z)-2,2'-[(1Z,2Z)-1,2-Ethanediylidene]dihydrazinecarboxamide | Present | Present | Absent | Absent | Absent | Absent |
|  | (3beta)-28-Hydroxy-28-oxoolean-12-en-3-yl 3-O-[(1S,2S)-2-carboxy-1-(carboxymethoxy)-2-hydroxyethyl]-beta-D-glucopyranosiduronic acid | Present | Present | Absent | Absent | Absent | Absent |
|  | 1-Hexadecanoylpyrrolidine | Present | Present | Absent | Absent | Absent | Absent |
|  | 1-hexadecyl-2-amino-2-deoxy-sn-glycerol | Present | Present | Absent | Absent | Absent | Absent |
|  | 1-Isothiocyanato-15-(methylsulfanyl)pentadecane | Present | Present | Absent | Absent | Absent | Absent |
|  | 1-Morpholinocyclohexene | Present | Present | Absent | Absent | Absent | Absent |
|  | 1-Naphthaleneacetic acid | Present | Present | Absent | Absent | Absent | Absent |
|  | 1-Naphthol-4-sulfonic acid | Present | Present | Absent | Absent | Absent | Absent |
|  | 1-Nitrosopyrene | Present | Present | Absent | Absent | Absent | Absent |
|  | 1-Octyl-3,5-bis(octyloxy)-4-piperidinol | Present | Present | Absent | Absent | Absent | Absent |
|  | (4-Ethoxyphenyl)urea | Present | Present | Absent | Absent | Absent | Absent |
|  | (4R)-4-{[(2S)-2-Acetamido-3-carboxypropanoyl]amino}-5-{[(2S)-1-({(2S)-1-[(2S,4R)-4-(benzyloxy)-2-{[2-(2,3-dihydro-1H-indol-1-ylcarbonyl)-2-propylhydrazino]carbonyl}-1-pyrrolidinyl]-3-methyl-1-oxo-2-bu tanyl}amino)-3-methyl-1-oxo-2-pentanyl]amino}-5-oxopentanoic acid | Present | Present | Absent | Absent | Absent | Absent |
|  | (4R,6R)-t-Butyl-6-(2-aminoethyl)-2,2-dimethyl-1,3-dioxane-4-acetate | Present | Present | Absent | Absent | Absent | Absent |
|  | 1-t-butyl-4-ethylbenzene | Present | Present | Absent | Absent | Absent | Absent |
|  | [4,6-Bis(isopropylamino)-1,3,5-triazin-2-yl]cyanamide | Present | Present | Absent | Absent | Absent | Absent |
|  | 2-(2-Pyridinyl)-5-pyrimidinecarboxylic acid | Present | Present | Absent | Absent | Absent | Absent |
|  | 2-(9H-Fluoren-2-ylcarbonyl)benzoic acid | Present | Present | Absent | Absent | Absent | Absent |
|  | 2-(Adamantan-1-ylamino)ethanol | Present | Present | Absent | Absent | Absent | Absent |
|  | 2-(Didodecylamino)ethanol | Present | Present | Absent | Absent | Absent | Absent |
|  | 2-(Diethylamino)-6-methyl-4(1H)-pyrimidinone | Present | Present | Absent | Absent | Absent | Absent |
|  | 2-(N'-Cyano-N''-methylcarbamimidamido)ethyl methanesulfonate | Present | Present | Absent | Absent | Absent | Absent |
|  | 2,2'-(1,5-Pentanediyldiimino)diethanol | Present | Present | Absent | Absent | Absent | Absent |
|  | 2,2'-(4-Cyclohexene-1,2-diyl)diacetonitrile | Present | Present | Absent | Absent | Absent | Absent |
|  | 1,2,4,5-Tetraazaspiro[5.5]undecane-3-thione | Present | Present | Absent | Absent | Absent | Absent |
|  | 1,2,4-Tribromo-9-chloro-3H-phenothiazin-3-one | Present | Present | Absent | Absent | Absent | Absent |
|  | 2,2,6,6-Tetramethyl-4-piperidyl Methacrylate | Present | Present | Absent | Absent | Absent | Absent |
|  | 2,2'-[(12-Methyl-1,12-tridecanediyl)bis(oxy)]diethanol | Present | Present | Absent | Absent | Absent | Absent |
|  | 1,3-Dicyclohexylurea | Present | Present | Absent | Absent | Absent | Absent |
|  | 2,2'-[(Octadecylimino)bis(2,1-ethanediyloxy)]diethanol | Present | Present | Absent | Absent | Absent | Absent |
|  | 2,2'-{[3-(Dodecyloxy)propyl]imino}diethanol | Present | Present | Absent | Absent | Absent | Absent |
|  | 2,3-Dihydroxypropyl stearate | Present | Present | Absent | Absent | Absent | Absent |
|  | 151L | Present | Present | Absent | Absent | Absent | Absent |
|  | 2,5,8,11,14-Pentaoxatetracosane | Present | Present | Absent | Absent | Absent | Absent |
|  | 2,5-Bis(heptylamino)-3,6-diphenyl-1,4-benzoquinone | Present | Present | Absent | Absent | Absent | Absent |
|  | 2,5-Bis(tert-butylperoxy)-2,5-dimethylhexane | Absent | Present | Absent | Absent | Absent | Absent |
|  | 2,6-Bis-(acetamido)-pyridine | Present | Present | Absent | Absent | Absent | Absent |
|  | 2,6-Difluoro-3-pyridinol | Present | Present | Absent | Absent | Absent | Absent |
|  | 1-Methyl-1,2,3,4-tetrahydro-Î²-carboline-3-carboxylic acid | Present | Present | Absent | Absent | Absent | Absent |
|  | 2-[(2-Bromo-2-propen-1-yl)sulfanyl]ethanol | Present | Present | Absent | Absent | Absent | Absent |
|  | 2-((4-Nitrophenoxy)methyl)oxirane | Present | Present | Absent | Absent | Absent | Absent |
|  | 2-[(6-{[(5-Fluoro-2,4-dioxo-3,4-dihydro-1(2H)-pyrimidinyl)carbonyl]amino}hexanoyl)oxy]-1,3-propanediyl didecanoate | Present | Present | Absent | Absent | Absent | Absent |
|  | 2-[(N-Benzoylphenylalanyl)amino]-3-phenylpropyl acetate | Present | Present | Absent | Absent | Absent | Absent |
|  | 2-[4'-(Hexyloxy)-4-biphenylyl]-5-pyrimidinyl octanoate | Present | Present | Absent | Absent | Absent | Absent |
|  | 2-{1-[2-(2,3-Dihydro-1-benzofuran-5-yl)ethyl]-3-pyrrolidinyl}-2,2-diphenylacetamide | Present | Present | Absent | Absent | Absent | Absent |
|  | 2-{3-[4-(2-Hydroxyethyl)-1-piperazinyl]propyl}-6-methyl-4-(1-piperidinyl)-1H-pyrrolo[3,4-c]pyridine-1,3(2H)-dione | Present | Present | Absent | Absent | Absent | Absent |
|  | 26-Acetamido-22-oxocholest-5-ene-3,16-diyl diacetate | Present | Present | Absent | Absent | Absent | Absent |
|  | 2,2'-(Tridecylimino)diethanol | Present | Present | Absent | Absent | Absent | Absent |
|  | 2-Amino-1,3,4-octadecanetriol | Present | Present | Absent | Absent | Absent | Absent |
|  | 2-Amino-2,5-dihydro-1,5,2-diazaphosphinin-6(1H)-one 2-oxide | Present | Present | Absent | Absent | Absent | Absent |
|  | 2-amino-3-cyanoquinoline | Present | Present | Absent | Absent | Absent | Absent |
|  | 2-Amino-4-pentynoic acid | Absent | Present | Absent | Absent | Absent | Absent |
|  | 2,2,8,8-Tetramethyloctahydro[1,2,4,3]triazaphosphinino[3,4-c][1,2,4,3]triazaphosphinine-2,8-diium 10-oxide | Present | Present | Absent | Absent | Absent | Absent |
|  | 2,2'-[(4,6-Diamino-1,3,5-triazin-2-yl)imino]diethanol | Present | Present | Absent | Absent | Absent | Absent |
|  | 2-Chloro-N-(2-chloroethyl)-N-(4-ethylbenzyl)ethanamine | Present | Present | Absent | Absent | Absent | Absent |
|  | 2-Cyclododecyl-1-(4-morpholinyl)ethanone | Present | Present | Absent | Absent | Absent | Absent |
|  | 2,4,6-Tri-tert-butylphenol | Present | Present | Absent | Absent | Absent | Absent |
|  | 2-Hexadecylpyridine | Absent | Present | Absent | Absent | Absent | Absent |
|  | 2,6-Dichloro-4-nitrophenol | Present | Present | Absent | Absent | Absent | Absent |
|  | 2,6-di-tert-butyl-4-ethylphenol | Present | Present | Absent | Absent | Absent | Absent |
|  | 2-[(2-Hydroxyethyl)(stearoyl)amino]ethyl stearate | Present | Present | Absent | Absent | Absent | Absent |
|  | 2-Methyl-2-propanyl (3S)-3-{[(2-methoxyphenyl)sulfonyl]amino}-1-piperidinecarboxylate | Present | Present | Absent | Absent | Absent | Absent |
|  | 2-Methyl-2-propanyl [(2S,3R)-1,3-dihydroxy-2-octadecanyl]carbamate | Present | Present | Absent | Absent | Absent | Absent |
|  | 2-Naphthalenesulfonic acid | Present | Present | Absent | Absent | Absent | Absent |
|  | 2-Oxaziridinesulfonic acid | Present | Present | Absent | Absent | Absent | Absent |
|  | 2-Trifluoromethylpyrrolidine | Present | Present | Absent | Absent | Absent | Absent |
|  | 3-(1-Boc-4-piperidyl)-1-propanol | Present | Present | Absent | Absent | Absent | Absent |
|  | 2-Benzoyl-1,3-diphenyl-1,3-propanedione | Present | Present | Absent | Absent | Absent | Absent |
|  | 3'-(4-Fluorophenyl)-3,4-dihydro-1H-spiro[naphthalene-2,2'-oxiran]-1-one | Present | Present | Present | Absent | Absent | Absent |
|  | 3-(Dodecylamino)-1,2-propanediol | Present | Present | Present | Absent | Absent | Absent |
|  | 3,3'-(dodecylimino)bispropane-1,2-diol | Absent | Present | Present | Absent | Absent | Absent |
|  | 2-Hydroxy-5-(1,1,3,3-tetramethylbutyl)benzenesulfonic acid | Present | Present | Present | Absent | Absent | Present |
|  | 3,3-Bis(phenylsulfanyl)-4-undecanol | Present | Present | Present | Absent | Absent | Present |
|  | 3,3-Dicyclohexylalanine | Present | Present | Present | Absent | Present | Present |
|  | 2-Hydroxy-N,N,N-trimethyl-4-oxo-4-[2-(trimethylammonio)ethoxy]-1-butanaminium | Present | Present | Present | Absent | Present | Present |
|  | 3,4-Dihexadecyl-2-hydroxybenzoic acid | Present | Present | Present | Present | Present | Present |
|  | 3,6,9,12,15,18,21,24-Octaoxaheptatriacontan-1-ol | Present | Present | Present | Present | Present | Present |
|  | 3,6,9,12,15,18,21-Heptaoxadotriacontan-1-ol | Present | Present | Present | Present | Present | Present |
|  | 3,6,9,12,15,18-Hexaoxahentriacontan-1-ol | Present | Present | Present | Present | Present | Present |
|  | 3,6,9,12-Tetraoxapentacosan-1-ol | Present | Present | Present | Present | Present | Present |
|  | 3',6'-Dihydroxy-3H-spiro[2-benzofuran-1,9'-thioxanthen]-3-one | Present | Present | Present | Present | Present | Present |
|  | 3,7,11-Tris(2-hydroxyethyl)-15-oxa-3,7,11-triazaheptacosane-1,13-diol | Present | Present | Present | Present | Present | Present |
|  | 3-[(4,6-Diamino-1,3,5-triazin-2-yl)amino]-1-propanol | Present | Present | Present | Present | Present | Present |
|  | 3-[Tris(decyloxy)silyl]-1-propanethiol | Present | Present | Present | Present | Present | Present |
|  | 3-Fluoroaspartic acid | Present | Present | Present | Present | Present | Present |
|  | 3-hexadecyloxy propylamine | Present | Present | Present | Present | Present | Present |
|  | 3-Mercapto-3-methylbutan-1-ol | Absent | Present | Present | Present | Present | Present |
|  | 3-Phosphonooxypyruvic acid | Present | Present | Present | Present | Present | Present |
|  | 3-(Octadecylamino)-1,2-propanediol | Present | Present | Present | Present | Present | Present |
|  | 4-(4-Hydroxy-4-methylpentyl)cyclohex-3-ene-1-carbaldehyde | Present | Present | Present | Present | Present | Present |
|  | 4-(4-Methoxyphenyl)-2-thiophenecarboxylic acid | Present | Present | Present | Present | Present | Present |
|  | 3-(tetradecyloxy)propylamine | Present | Present | Present | Present | Present | Present |
|  | 4-(Dioctadecylamino)-4-oxobutanoic acid | Present | Present | Present | Present | Present | Present |
|  | 4-(Dioxido-lambda~6~-sulfanylidene)-2,5-cyclohexadien-1-imine | Present | Present | Present | Present | Present | Present |
|  | 4-(Methylsulfanyl)-3-phenylspiro[thiete-2,9'-xanthene] | Present | Present | Present | Present | Present | Present |
|  | 4,4'-Methylenebis(6-methyl-1,3-benzenediamine) | Present | Present | Present | Present | Present | Present |
|  | 4,4'-Oxydianiline | Present | Present | Present | Present | Present | Present |
|  | 4,5,6,7-Tetrachloro-3-nitroso-2H-isoindol-1-amine | Present | Present | Present | Present | Present | Present |
|  | 4,7,10,13,16,19,22-Heptaoxapentacosane-1,25-diamine | Present | Present | Present | Present | Present | Present |
|  | 3,5-Bis{[bis(2-methyl-2-propanyl)phosphino]methyl}-2,4,6-trimethylphenol | Present | Present | Present | Present | Present | Present |
|  | 4-Amino-2,6-dichlorophenol | Present | Present | Present | Present | Present | Present |
|  | 3-Hydroxy-2-naphthydrazide | Present | Present | Present | Present | Present | Present |
|  | 4-Dodecanoyl-2,6-piperazinedione | Present | Present | Present | Present | Present | Present |
|  | 4-Dodecylbenzenesulfonic acid | Present | Present | Present | Present | Present | Present |
|  | 4-Hydrazinobenzenesulfonic acid | Present | Present | Present | Present | Present | Present |
|  | 4-Hydroxy-2-oxo-1,3,2-dioxaphosphetan-2-ium | Present | Present | Present | Present | Present | Present |
|  | 3-Methoxy-7-oxo-7H-benzo[de]anthracene-9-sulfonic acid | Present | Present | Present | Present | Present | Present |
|  | 4,7,10,13,16,19,22,25,28,31,34,37-Dodecaoxahexatetracontane | Present | Present | Present | Present | Present | Present |
|  | 4-Nonanoylmorpholine | Present | Present | Present | Present | Present | Present |
|  | 4-tert-Butylphenyl Salicylate | Present | Present | Present | Present | Present | Present |
|  | 4-Undecylbenzenesulfonic acid | Present | Present | Present | Present | Present | Present |
|  | 5-(4-Butoxy-3-ethoxyphenyl)-1-[3-(diethylamino)propyl]-4-[hydroxy(4-pyridinyl)methylene]-2,3-pyrrolidinedione | Present | Present | Present | Present | Present | Present |
|  | 5-(Ethoxymethyl)-2-methyl-4-pyrimidinamin | Absent | Present | Present | Present | Present | Present |
|  | 5,6-Dimethoxy-1-benzothiophene-2-carboxylic acid | Present | Present | Present | Present | Present | Present |
|  | 5-Butyl-4-phenyl-2-pyrimidinamine | Present | Present | Present | Present | Present | Present |
|  | 5-Chloro-3-fluoro-2-pyridinol | Present | Present | Present | Present | Present | Present |
|  | 5-Hydroxy-DL-tryptophan | Present | Present | Present | Present | Present | Present |
|  | 5-Methyl-4,6-dioxohexahydro-2-pyrimidinesulfonic acid | Present | Present | Present | Present | Present | Present |
|  | 5-(Dimethylamino)-4,4-dimethyl-1,1,5-triphenyl-1-pentanol | Present | Present | Present | Present | Present | Present |
|  | 5-Boc-Octahydropyrrolo[3,4-c]pyridine | Present | Present | Present | Present | Present | Present |
|  | 8-Hydroxy-1-[4-hydroxy-3-(~125~I)iodophenyl]-8-oxido-3,14-dioxo-7,9,13-trioxa-4-aza-8lambda~5~-phosphanonacosan-11-yl palmitate | Present | Present | Present | Present | Present | Present |
|  | 9,10-Dihydroxystearic acid | Absent | Present | Present | Present | Present | Present |
|  | 5-Octadecyl-2,4,6-pyrimidinetriamine | Present | Present | Present | Present | Present | Present |

**Table S3.** Details about the remaining concentrations of BOD, COD, Sulphate and Phosphate in the FTB treated samples.

| **BOD remaining after FTB treatment in mg/L** | | | | | | | | | | |
| --- | --- | --- | --- | --- | --- | --- | --- | --- | --- | --- |
| **FTBs** | **1 day** | | **2 day** | | **3 day** | | **5 day** | | **10 day** | |
|  | **Mean** | **SD** | **mean** | **SD** | **mean** | **SD** | **mean** | **SD** | **mean** | **SD** |
| FT- P-CN | 33.12 | 0.32 | 32.40 | 0.29 | 30.58 | 0.92 | 24.09 | 0.53 | 23.38 | 1.75 |
| FT-P-CI | 22.42 | 0.21 | 18.25 | 0.28 | 15.09 | 0.58 | 14.4 | 1.59 | 13.96 | 0.86 |
| FT-P-TL | 23.19 | 0.02 | 18.87 | 1.50 | 14.22 | 0.58 | 17.5 | 0.84 | 16.45 | 0.84 |
| FT-S-CN | 33.53 | 0.44 | 33.14 | 0.21 | 31.30 | 0.55 | 26.67 | 0.74 | 25.73 | 2.35 |
| FT-S-CI | 24.39 | 0.27 | 22.82 | 0.89 | 20.86 | 0.43 | 20.79 | 0.47 | 19.61 | 1.62 |
| FT-S-TL | 24.88 | 0.10 | 22.64 | 0.48 | 20.88 | 0.31 | 20.25 | 2.2 | 19.19 | 1.97 |
| FT-P-CI + VP3 | 19.52 | 0.21 | 16.38 | 0.28 | 11.44 | 0.58 | 7.99 | 1.59 | 7.21 | 0.86 |
| FT-P-TL + VP3 | 15.60 | 0.27 | 14.55 | 0.89 | 9.92 | 0.43 | 4.74 | 0.47 | 4.31 | 1.62 |
| FT-S-CI + VP3 | 34.53 | 0.10 | 34.31 | 0.48 | 32.87 | 0.31 | 28.88 | 2.23 | 28.38 | 1.97 |
| FT-S-TL + VP3 | 30.89 | 0.44 | 29.94 | 0.21 | 26.79 | 0.55 | 25.92 | 0.74 | 24.39 | 2.35 |
| **Phosphate remaining after FTB treatment in mg/L** | | | | | | | | | | |
| FT- P-CN | 107.98 | 0.55 | 95.62 | 0.93 | 112.44 | 0.13 | 108.03 | 2.12 | 102.46 | 0.81 |
| FT-P-CI | 40.99 | 0.28 | 69.75 | 0.50 | 58.79 | 0.13 | 65.89 | 2.01 | 47.98 | 0.24 |
| FT-P-TL | 36.71 | 0.09 | 67.33 | 0.20 | 58.01 | 0.12 | 61.67 | 0.76 | 44.95 | 0.62 |
| FT-S-CN | 106.61 | 1.64 | 95.02 | 0.71 | 109.22 | 0.24 | 109.01 | 2.51 | 102.00 | 1.12 |
| FT-S-CI | 80.14 | 0.24 | 73.02 | 0.18 | 61.77 | 0.15 | 56.40 | 0.91 | 58.29 | 1.17 |
| FT-S-TL | 78.51 | 0.49 | 75.68 | 0.76 | 60.84 | 0.13 | 60.04 | 0.62 | 55.38 | 0.67 |
| FT-P-CI + VP3 | 40.84 | 0.09 | 36.45 | 0.20 | 34.10 | 0.12 | 44.66 | 0.76 | 20.5 | 0.62 |
| FT-P-TL + VP3 | 32.89 | 1.64 | 28.80 | 0.71 | 26.78 | 0.24 | 24.03 | 2.51 | 18.20 | 1.12 |
| FT-S-CI + VP3 | 45.99 | 0.49 | 43.22 | 0.76 | 34.66 | 0.13 | 45.76 | 0.62 | 27.48 | 0.67 |
| FT-S-TL + VP3 | 44.71 | 0.09 | 42.40 | 0.20 | 34.09 | 0.12 | 47.75 | 0.76 | 24.25 | 0.62 |
| **COD remaining after FTB treatment in mg/L** | | | | | | | | | | |
| FT- P-CN | 843.69 | 0.53 | 825.33 | 0.14 | 778.9 | 0.72 | 685.56 | 0.90 | 676.57 | 0.90 |
| FT-P-CI | 667.46 | 0.25 | 677.84 | 0.83 | 471.57 | 0.72 | 490.88 | 0.55 | 460.93 | 0.64 |
| FT-P-TL | 581.80 | 0.95 | 543.62 | 1.05 | 488.19 | 0.30 | 464.11 | 0.018 | 464.14 | 0.54 |
| FT-S-CN | 854.27 | 0.74 | 844.32 | 0.21 | 797.47 | 0.20 | 706.41 | 0.8 | 700.42 | 0.86 |
| FT-S-CI | 633.01 | 1.68 | 611.00 | 1.15 | 561.23 | 0.88 | 559.34 | 1.93 | 529.37 | 2.53 |
| FT-S-TL | 702.18 | 0.56 | 690.15 | 0.52 | 528.43 | 0.22 | 539.30 | 0.23 | 512.34 | 1.64 |
| FT-P-CI + VP3 | 351.58 | 0.5 | 359.65 | 0.14 | 206.85 | 0.72 | 182.42 | 0.9 | 165.70 | 0.90 |
| FT-P-TL + VP3 | 236.77 | 0.25 | 210.71 | 0.83 | 174.67 | 0.72 | 144.05 | 0.55 | 155.91 | 0.64 |
| FT-S-CI + VP3 | 337.13 | 0.95 | 296.95 | 1.05 | 269.82 | 0.30 | 260.85 | 0.018 | 243.78 | 0.54 |
| FT-S-TL + VP3 | 367.04 | 0.74 | 380.158 | 0.21 | 228.58 | 0.20 | 217.63 | 0.8 | 200.7 | 0.86 |
| **Sulphate remaining after FTB treatment in mg/L** | | | | | | | | | | |
| FT- P-CN | 839.54 | 13.28 | 824.01 | 3.67 | 735.72 | 17.82 | 728.45 | 22.31 | 641.25 | 22.31 |
| FT-P-CI | 342.09 | 6.31 | 322.9 | 20.68 | 264.81 | 17.94 | 119.82 | 13.71 | 238.99 | 15.9 |
| FT-P-TL | 300.22 | 23.70 | 253.56 | 26.08 | 170.48 | 7.51 | 104.13 | 0.44 | 223.30 | 13.52 |
| FT-S-CN | 845.55 | 18.46 | 830.48 | 5.4 | 765.44 | 5.18 | 792.68 | 19.99 | 635.72 | 21.29 |
| FT-S-CI | 456.1 | 41.72 | 387.53 | 28.52 | 323.38 | 21.84 | 167.12 | 47.79 | 332.80 | 62.78 |
| FT-S-TL | 328.7 | 14.07 | 299.68 | 12.9 | 227.16 | 5.61 | 182.91 | 5.88 | 328.25 | 40.79 |
| FT-P-CI + VP3 | 350.13 | 13.28 | 336.04 | 3.6 | 142.44 | 17.82 | 46.34 | 22.31 | 90.22 | 22.31 |
| FT-P-TL + VP3 | 328.19 | 6.31 | 307.24 | 20.68 | 27.18 | 17.94 | 21.40 | 13.71 | 114.54 | 15.93 |
| FT-S-CI + VP3 | 432.11 | 23.70 | 415.55 | 26.08 | 165.98 | 7.51 | 143.2 | 0.44 | 174.87 | 13.52 |
| FT-S-TL + VP3 | 337.76 | 18.46 | 328.17 | 5.40 | 44.07 | 5.18 | 37.70 | 19.99 | 184.90 | 21.29 |

**Table S4.** ARG profiling of the Mini river water after VP3 incorporated FTBs treatment**.** Where, ✓ = present and 🗶 = absent. FT represents Floating bed treatment, P stands for Polystyrene frame, S stands for styrofoam frame, CN stands for control, CI stands for *C. indica*, and TL stands for *T. latifolia*.

| **FTBs**  **ARGs** | FT-P-CN | FT-P-CI | FT-P-TL | FT-S-CN | FT-S-CI | FT-S-TL |
| --- | --- | --- | --- | --- | --- | --- |
| *blaTEM* |  |  |  |  |  |  |
| *sul1* |  |  |  |  |  |  |
| *sul2* |  |  |  |  |  |  |
| aac (6’)-Ib-Cr |  |  |  |  |  |  |
